# Autologous platelet concentrates as adjuncts to non-surgical periodontal therapy: a systematic review and meta-analysis

**DOI:** 10.1007/s00784-024-06128-w

**Published:** 2025-01-22

**Authors:** Tina Lipovec, N. Kapadia, G. N. Antonoglou, E. M. C. Lu, K. M. Fawzy El-Sayed, Luigi Nibali

**Affiliations:** 1 Periodontology Unit, Centre for Host Microbiome Interactions, Faculty of Dentistry, Oral & Craniofacial Sciences, King’s College London, London, UK; 2 Interdisciplinary Doctoral Degree Programme Biomedicine, Clinical Medicine, Faculty of Medicine, University of Ljubljana, Ljubljana, Slovenia; 3 Oral Medicine and Periodontology Department, Faculty of Dentistry, Cairo University, Cairo, Egypt; 4 Stem Cells & Tissue Engineering Unit, Faculty of Dentistry, Cairo University, Cairo, Egypt; 5 Clinic for Conservative Dentistry and Periodontology, School of Dental Medicine, Christian Albrechts University, Kiel, Germany

**Keywords:** Autologous, Platelet, Periodontitis, Meta-analysis, Non-surgical periodontal therapy, Professional mechanical plaque removal

## Abstract

**Objective:**

To evaluate the possible additional clinical benefit from autologous platelet concentrate (APC) treatment adjunct to non-surgical periodontal therapy (NSPT).

**Methods:**

Electronic (MEDLINE/Embase/Cochrane/MedNar/CORE) and hand searches were conducted. Following studies selection, evidence tables were formed, and meta-analyses were performed for the following outcomes: probing pocket depth (PPD) reduction, clinical attachment level (CAL) gain, and bleeding on probing (BoP) reduction. The protocol for this systematic review was registered in PROSPERO (CRD42023514388).

**Results:**

After de-duplication, the initial search yielded 194 citations, from which ten papers were eligible for quantitative synthesis. The APC group comprised 270 patients, while the control group included 230. The meta-analysis revealed that a single APC application resulted in a 0.6 mm greater PPD reduction (MD = -0.62; 95% CI: -1.03, -0.22) and 0.8 mm more CAL gain (MD = -0.77; 95% CI: -1.18, -0.37) at the 6–12 weeks follow-up. At six months, the APC group exhibited a 0.6 mm greater PPD reduction (MD = -0.61; 95% CI: -1.13, -0.09) and 1.1 mm more CAL gain (MD = -1.14; 95% CI: -1.94, -0.34) compared to the NSPT only group. In contrast, BoP indices did not reveal a statistically significant difference between the groups after 6–12 weeks (MD = -10.54; 95% CI: -25.21, 4.14). High heterogeneity and unclear to high risk of bias were detected.

**Conclusion:**

Over six months, the adjunctive APC use appears to provide additional benefits in PPD reduction and CAL gain compared to NSPT alone.

**Clinical relevance:**

The adjunctive use of APCs seems to promote further improvements in clinical outcomes following NSPT.

**Supplementary Information:**

The online version contains supplementary material available at 10.1007/s00784-024-06128-w.

## Introduction

Non-surgical periodontal therapy (NSPT), recently defined as professional mechanical plaque removal [1], irrespective of the type of instruments used, alters the subgingival ecological environment by removing calculus deposits and disrupting the microbial biofilm, thereby reducing soft-tissue inflammation and promoting wound healing [1, 2]. The periodontal treatment endpoints of favourable treatment response are defined as an absence of residual pockets with probing pocket depth (PPD) > 4 mm with bleeding on probing (BoP) and a complete absence of residual pockets with PPD ≥ 6 mm as surrogate measures of healing of the periodontium [3]. Pocket closure occurs in approximately three-quarters of sites after NSPT [1], indicating surgical interventions may be necessary in the remaining quarter of periodontal pockets, thereby imposing additional biological and financial burdens on the patient. Thus, there is a pressing clinical need to improve non-surgical treatment outcomes over the current gold standard of NSPT. This highlights the need for the incorporation of adjunctive therapies to address the current shortcomings [4–8].

It is traditionally believed that healing by repair [9] occurs after NSPT, with histological evidence of alveolar bone repair and formation of the long junctional epithelium (LJE) [10]. As an alternative to repair, the concept of periodontal regeneration aims to restore the original structure and function of the lost tissues by reactivating fundamental healing mechanisms employed in their formation.

Autologous platelet concentrates (APCs) are biologics obtained from the individual’s own blood that contain highly elevated platelet concentrations, mimicking the coagulation cascade’s end stage [11]. They are generated by the centrifugation of venous whole blood at varying speeds, with or without the inclusion of exogenous substances. Depending on the preparation technique, there are 3 main categories of APCs: platelet-rich plasma (PRP) [12], platelet-rich fibrin (PRF) [13], and concentrated growth factors (CGF) [14].

APCs, abundant in growth factors (GFs), cytokines, and other bioactive molecules, play an essential role in creating a favourable wound healing environment by actively engaging in critical phases of the healing process. The platelets’ release of GFs stimulates cell recruitment, proliferation, differentiation, angiogenesis, and extracellular matrix synthesis, which acts as a scaffold for cell migration and provides structural support during the healing process [15–20]. Furthermore, their anti-inflammatory properties help to modulate the immune response at the treatment site, promoting tissue remodeling and maturation [17, 21, 22].

Numerous studies have demonstrated the efficacy of APCs as a regenerative tool for reconstructing both hard and soft tissues in patients suffering from periodontal disease, mostly utilizing them in surgical procedures [21, 23–29]. These promising results have prompted the investigation into utilizing APCs as an adjunctive modality in NSPT [30–35]. As there is a need to try and maximize the efficacy of non-surgical periodontal therapy, it is important to assess the potential role of different APC subtypes and APC as a whole as adjuncts to NSPT. Therefore, the aim of this systematic review and meta-analysis was to assess the clinical effects of APCs when used as an adjunctive treatment in NSPT.

## Materials and methods

### Protocol development and focused question

The protocol was co-designed by all authors and registered in PROSPERO on 29 February 2024 under ID CRD42023514388. No protocol amendments have been made after its registration. This review was reported using the Preferred Reporting Items for Systematic Review and Meta-Analyses (PRISMA 2020) guidelines [36]; the PRISMA statement is attached in Appendix 1.

The primary objective of this systematic review was to assess the potential role of APCs in the outcomes of NSPT by answering the following focused question: “Do autologous platelet concentrates provide beneficial effects when used as adjuncts to non-surgical periodontal therapy?”.

The research question was formulated based on the following PICOST criteria:

- Population: Adult patients with periodontitis.
- Intervention: NSPT (standard, modified, hand or powered) with adjunctive APCs.
- Comparison: NSPT (standard, modified, hand, or powered) without adjuncts.
- Outcomes:
  - Primary: Probing Pocket Depth (PPD) reduction.
  - Secondary: Clinical Attachment Level (CAL) gain, Gingival Recession Depth (RD), Bleeding on Probing (BoP) reduction, pocket closure (defined as PPD ≤ 4 mm without BoP or PPD ≤ 3 mm solely), radiographic parameters (radiographically linear detected depth (RLDD) and radiographic bone fill), local and systemic inflammatory changes (e.g., cytokine levels in GCF, saliva, circulation), patient-reported outcomes (PROMs).
- Type of studies: Randomized controlled trials (RCTs).
- Timing: ≥ 6 weeks after NSPT.

### Eligibility criteria

Studies were included if they met the following eligibility criteria:

(i) Study design: RCTs.
(ii) Studies including 10 or more test subjects.
(iii) Adult participants (≥ 18 years old) diagnosed with periodontitis and have undergone NSPT, with one arm having adjunctive APCs.
(iv) Includes full-mouth periodontal parameters after NSPT, such as:
  - PPD,
  - RD,
  - CAL,
  - BoP,
  - Percentage or number of ‘closed pockets’ (PPD ≤ 3 mm or PPD ≤ 4 mm without BoP) out of the total number of pockets at baseline (PPD > 3 mm or PPD > 4 mm),
  - Changes in the percentage of pockets before and after NSPT,
  - The mean number of residual pockets after NSPT,
  - Radiographic outcomes.
(v) Follow-up time of at least 6 weeks.

### Definitions

#### Definition of 'non-surgical periodontal therapy’

Within the context of this review, ’non-surgical periodontal therapy’ was defined as the disruption of the microbial biofilm and removal of soft and hard subgingival deposits, recently defined as professional mechanical plaque removal [1], but previously also known as scaling and root planning (SRP), root surface debridement, subgingival scaling, etc., without any restrictions on the type of instruments used and the mode of delivery.

#### Definition of ‘intrabony defects’ and ‘radiographically linear defect depth (RLDD)’

Intrabony defects, which may also be referred to as vertical or angular defects, were defined as periodontal defects within the bone, surrounded by one, two, or three bony walls or a combination thereof [37]. Although this systematic review will not focus solely on intrabony defects, this definition will allow differentiation of studies focusing on intrabony defects from others, including a combination of other types of periodontal defects.

RLDD was defined as the radiographically linear detected depth of the intrabony defect, which, in different studies, may be measured from the alveolar crest or the cemento-enamel junction (CEJ) to the defect base. Because of differences in definitions between various studies, the present study will focus solely on the difference in RLDD before and after NSPT delivery.

#### Definition of ‘autologous platelet concentrates’

For this review, they were defined as autologous platelet concentrates prepared by obtaining the patient’s own blood and chair-side centrifugation (liquid or solid forms of platelet-rich fibrin (PRFs), platelet-rich plasma (PRP), plasma rich in growth factors (PRGF), and concentrated growth factors (CGF)).

### Search methods

The search strategy aimed to ensure comprehensive coverage by combining controlled vocabulary (MeSH terms) and free-text terms. It was then tested for a balance between sensitivity and precision, focusing on broad search coverage without any language restrictions in the initial search. Electronic databases, including MEDLINE (Ovid), Embase (Ovid), Cochrane Central Register of Controlled Trials (CENTRAL), MedNar, and CORE, were queried up to February 26, 2024, with no limitations on publication year. The search strategies for all electronic databases are presented in Appendix 2. Related narrative and systematic reviews were also searched and reviewed to identify suitable papers. Search results from all databases were combined, and duplicates were removed. To maximize the inclusion of relevant studies, the bibliographies of papers that matched the eligibility criteria were also screened to identify any further relevant references, which were then subject to the same screening and selection process.

### Study selection

The citations identified from the literature searches and reference list checking were imported to EndNote 21 (Clarivate, Philadelphia, USA), and duplicates were identified and manually removed. Two independent reviewers (authors TL and NK) conducted the study selection in two stages. In the first stage, potentially relevant papers were identified by screening all titles and abstracts against the inclusion criteria. This resulted in a complete database by merging studies included by at least one reviewer. In the second stage, full-text versions of papers identified as possibly relevant were retrieved and screened against the inclusion criteria; studies not meeting the inclusion criteria were excluded. In the presence of disagreement between reviewers, the decision regarding study eligibility was attempted by discussion. If consensus could not be reached, an arbitrator (author LN) judged the study’s inclusion. The level of agreement between reviewers was calculated using kappa statistics for the first- and second-stage screening.

### Data collection

A data extraction form was developed to extract study characteristics. Two reviewers (authors TL and NK) worked independently to collect relevant data from the included studies in a duplicated form. If a study compared more than two arms fulfilling the inclusion criteria, the data from the arms of interest were extracted.

### Data items

The following data were extracted:

- Study characteristics: Author, year, source of publication, funding, study design, and setting (country, hospital, university, community, other).
- Population: The number of patients, patients’ demographics (age, gender, ethnicity, smoking, socioeconomic factors, body mass index/obesity), definition and diagnosis of periodontitis.
- Intervention: Types of instruments used for NSPT delivery (manual and/or powered), details of the type of APCs used and their application (single/multiple), and follow-up times.
- Outcomes: Periodontal clinical parameters before and after NSPT (PPD, RD, CAL, BoP), percentage or number of closed pockets (PPD ≤ 4 mm without BoP or PPD ≤ 3 mm) out of the total number of pockets at baseline (PPD > 3 mm or PPD > 4 mm), changes in the percentage of pockets before and after NSPT, mean number of residual pockets after NSPT, radiographic outcomes, local and systemic inflammatory changes (e.g., cytokine levels in GCF, saliva, circulation), and PROMs.

Data on study characteristics, population, and interventions were transferred into an evidence table to summarize the included studies.

The primary outcome was PPD reduction expressed in millimetres. Secondary outcomes included CAL gain, RD, and BoP. Additional consideration was given to radiographic parameters, local and systemic inflammatory changes, and PROMs. The number of time points at which the outcome was measured was not limited, and data were extracted for all time points. All data in the Excel spreadsheets were reviewed to assess their suitability for synthesis and were subsequently entered into R 4.2.3 (R Foundation for Statistical Computing, Vienna, Austria) for quantitative analysis.

### Risk of bias assessment

Two reviewers (TL and NK) independently and in duplicate assessed the risk of bias in all included studies using the revised RoB 2.0 tool recommended by the Cochrane Collaboration for assessing the risk of bias in randomized controlled trials [38]. Each study was graded according to five items (randomization, deviation from intended interventions, missing outcome data, measurement of the outcome, and selection of the reported result), and an overall score for risk of bias was assigned.

To evaluate the presence of publication bias, we considered generating and visually inspecting contour-enhanced funnel plots for the meta-analyses comprised of 10 or more studies [39].

### Effect measures and data synthesis

Meta-analysis was considered to assess the Mean Difference (MD) with the 95% Confidence Interval (CI) of the same continuous outcomes (PPD, CAL, GR) and dichotomous outcomes (BoP, pocket closure) across different studies in the presence of a sufficient number of similar studies (at least 3).

The type of APCs used and the length of follow-up were considered when interpreting study findings and determining which outcomes were sufficiently similar to be combined for synthesis. As APC subtypes have different compositions and mechanisms of action, their biological effects on the periodontium could differ. Furthermore, study settings and patient populations were expected to vary among studies; therefore, a random-effects model was deemed a priori more appropriate to capture variability and calculate the average distribution of treatment effects across studies [40] and a standard inverse variance estimate was chosen.

To assess the heterogeneity between studies, we inspected forest plots, examined tau^2^ (absolute heterogeneity) and I^2^ (relative inconsistency), and calculated uncertainty intervals for all heterogeneity estimates. We also evaluated the localization of heterogeneity within the forest plot and existing uncertainties. We used 95% prediction intervals to account for existing heterogeneity and to assist in the meta-analytical interpretation by presenting a range of possible future effects across various clinical settings [41]. No methods were utilized to address the missing data values. Sensitivity analyses were performed by excluding studies identified as having a high risk of bias from the meta-analysis. All analyses were performed using R 4.2.3 (R Foundation for Statistical Computing, Vienna, Austria) by a single researcher (author GNA) with open data provision [42]. Two-sided P-values and an alpha level of 5% were applied.

### Certainty of evidence

We reviewed the overall results for each outcome and assessed the level of certainty in the evidence as high, moderate, low, or very low, using the GRADEpro Guideline Development Tool (McMaster University and Evidence Prime, 2024; gradepro.org).

## Results

### Search results

**Fig. 1:**
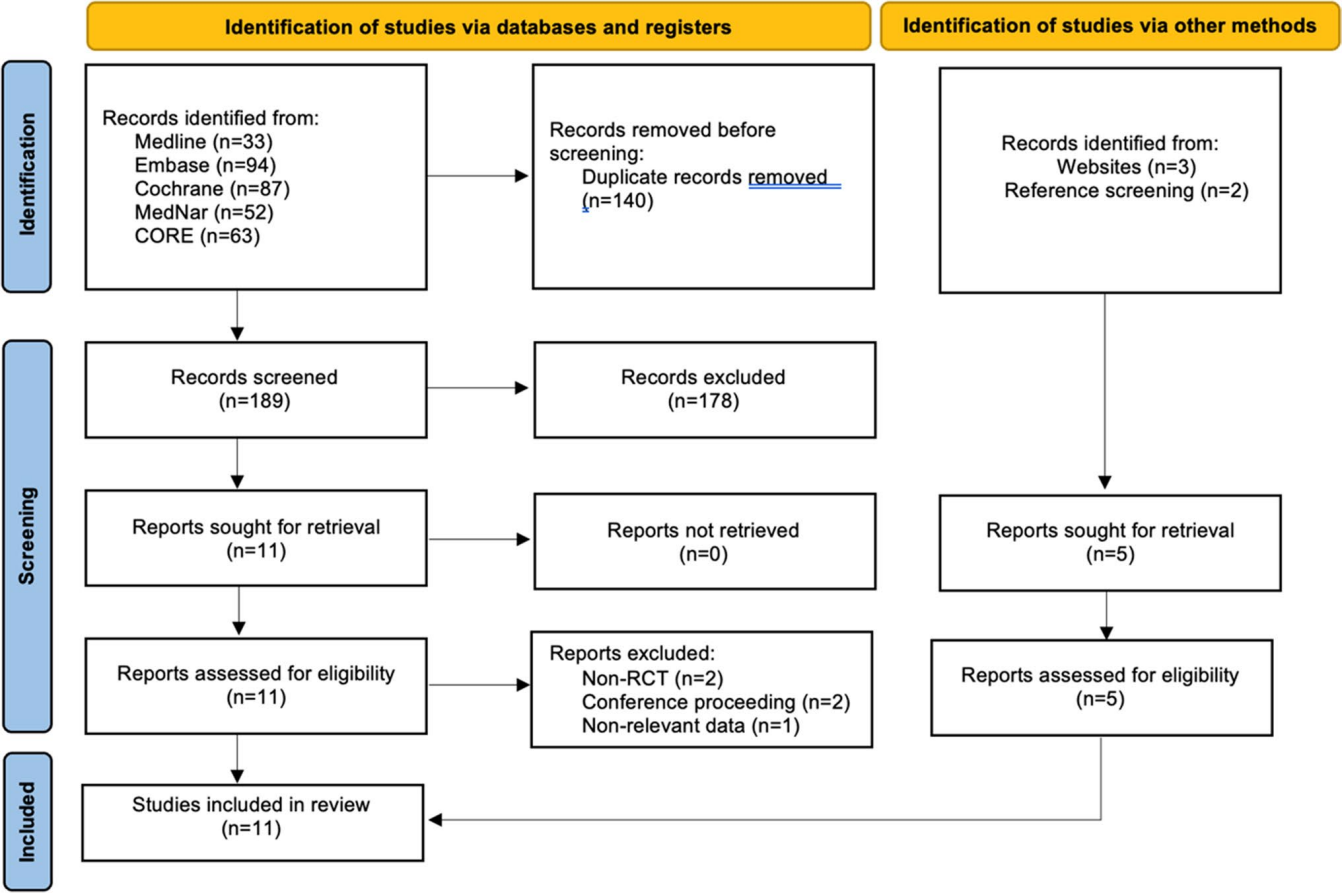
PRISMA 2020 Flow chart of study selection

The electronic and hand searches yielded 334 results. Duplicate removal resulted in 194 citations to be independently screened by title and abstract. The first screening stage led to the rejection of 178 papers, with the interrater agreement considered almost perfect, indicated by a kappa value of 0.87 (97.94%). 16 papers were eligible for full-text reading. Following the second screening stage, 11 papers were deemed eligible for inclusion in the review, with the interrater agreement again being deemed almost perfect at 93.75% and a kappa value of 0.85. The flow chart of study selection is illustrated in Fig. 1.

### Study characteristics

Table 1 provides descriptive summaries of the 11 included studies, listed chronologically by publication year and then alphabetically if published in the same year. Table 2, where the studies are listed by APC type and then chronologically where the same APC type was used, details the preparation and delivery of APCs along with post-operative instructions.

**Table 1:** Summary of characteristics of included studies

Author, year, country, title		Population	Treatment			Outcomes		Study conclusions
Agarwal & Dev Gupta (2015) India Platelet-rich Plasma with Scaling and Root Planing: A Double-blind Split-mouth Randomized Study		Setting: University (single center) *N* = *80* Age: 30–50 years Gender: Female and Male (*n* = *n/a*) Smoking: No Periodontal diagnosis: Moderate to severe CP Site selection (*n* = *160*): two contralateral single-rooted teeth with PPD ≥ 5 mm, corresponding angular bone defects ≥ 3 mm	RCT design: split-mouth Follow-up: 6 months NSPT delivery: full-mouth SRP APC type & application: PRP, single Test (*n* = *80*): SRP and PRP Control (*n* = *80)*: SRP and placebo gel			Values reported: Mean CAL, Mean PPD, Mean PI, Mean mSBI Timepoints: baseline, 3 and 6 months Other outcomes: /		PRP use during routine non-surgical debridement of periodontal pockets improves treatment outcomes in CP.
Panda et al. (2020) India Plasma rich in growth factors (PRGF) in non-surgical periodontal therapy: a randomized clinical trial		Setting: University (single center) *N* = *22* Age: 30–50 Gender: Female and Male (*n* = *n/a*) Smoking: < 10 cigarettes/day Periodontal diagnosis: Generalized stage III Grade A/B Site selection (*n* = *44*): two contralateral premolar or molar sites without FI and PPD ≥ 5 mm	RCT design: split-mouth Follow-up: 6 months NSPT delivery: full-mouth SRP, ultrasonic instrumentation, single session APC type & application: PRGF, single Test (*n* = *22*): SRP and PRGF Control (*n* = *22)*: SRP			Values reported: Mean PPD, Mean RAL, Mean SBI Mean PPD reduction Mean RAL gain Mean SBI change Pocket closure Timepoints: baseline, 3 and 6 months Other outcomes: /		The use of PRGF in NSPT by single intra-pocket application as an adjunct to SRP in CP patients was found to be effective in PPD reduction and CAL gain.
Vučković et al. (2020) Serbia The effect of injectable platelet-rich fibrin use in the initial treatment of chronic periodontitis		Setting: University (single center) *N* = *24* Age: 22–64 years (37 ± 10.23) Gender: Female *n* = *14*, Male *n* = *10* Smoking: No Periodontal diagnosis: CP Site selection (*n* = *not specified*): ≥ 6 teeth per quadrant; minimum of two contralateral teeth with PPD ≥ 5 mm; BoP ≥ 40%; all teeth and tooth sites, except third molars and degree II or III FI	RCT design: split-mouth Follow-up: 3 months NSPT delivery: full-mouth SRP under local anasthesia, one or two sessions in 24 h APC type & application: i-PRF, single Test (*n* = *not specified*): SRP and iPRF Control (*n* = *not specified*): SRP and saline			Values reported: Mean PPD, Mean CAL, Mean GML, Mean BoP, Mean PI Mean PPD decrease Mean CAL gain Mean GML decrease Timepoints: baseline, 3 months Other outcomes:/		Initial PT in conjunction with i-PRF proved to display significant improvements in all clinical parameters compared to initial PT alone.
Albonni et al. (2021) Syria Clinical effectiveness of a topical subgingival application of injectable platelet-rich fibrin as adjunctive therapy to scaling and root planing: a double-blind, split-mouth, randomized, prospective, comparative controlled trial		Setting: University (single center) *N* = *15* Age: 37–64 (45) Gender: Female *n* = *3*, Male *n* = *12* Smoking: *n* = *5* (10–20 cigarettes/day) Periodontal diagnosis: Stage II/III Grade B/C Site selection (*n* = *726*): bilateral pockets with PPD ≥ 5 mm, no degree II/III FI, tooth mobility, or plaque retention factors	RCT design: split-mouth Follow-up: 3 months NSPT delivery: full-mouth SRP under local anasthesia, ultrasonic and manual instrumentation APC type & application: i-PRF, single Test (*n* = *363*): SRP with i-PRF Control (*n* = *363*): SRP with saline			Values reported: Mean CAL, Mean PPD, Mean GR, Mean BoP, Full-mouth PI Pocket closure Timepoints: baseline and 3 months Other outcomes: /		The use of i-PRF as an adjunctive to mechanical SRP failed to result in additional improvement in terms of clinical indices compared to SRP alone.
Özcan et al. (2021) Turkey The effects of a novel non-invasive application of platelet-rich fibrin on periodontal clinical parameters and gingival crevicular fluid transforming growth factor-β and collagen-1 levels: A randomized, controlled, clinical study		Setting: University (single center) *N* = 12 Age: 43.33 ± 8.34 Gender: Female *n* = *6*, Male *n* = *6* Smoking: No Periodontal diagnosis: Stage III Grade B Site selection (*n* = *24*): 2 contralateral deep pockets in all teeth except for teeth with untreated caries, endodontic lesions, grade II mobility	RCT design: split-mouth Follow-up: 6 months NSPT delivery: full-mouth SRP, ultrasonic and manual instrumentation, single session APC type & application: PRF, single Test (*n* = *12*): SRP and PRF Control (*n* = *12*): SRP with saline		Values reported: Mean PI, Mean GI, Mean BoP, Mean PPD, Mean CAL, Mean GR Mean PPD reduction Mean CAL gain Mean GR increase Timepoints: baseline, 3 and 6 months Other outcomes: GCF Col-1 and TGF- β levels at baseline, 3rd, 7th, and 14th day		The adjunctive use of PRF with conventional SRP may effectively improve periodontal clinical parameters through increasing expression of the GCF Col-1 and TGF-β levels.	
Rakhewar et al. (2021) India An evaluation of clinical effects of i-PRF as an adjunctive to non-surgical periodontal therapy versus non-surgical periodontal therapy alone in treatment of chronic periodontitis patients – a split mouth randomized controlled clinical trial		Setting: University (single center) *N* = *10* Age: 30–50 years (39.2 ± 4.10) Gender: Female and Male (*n* = *n/a*) Smoking: No Periodontal diagnosis: Moderate to severe CP Site selection (*n* = *20*): minimum 2 contralateral sites with PPD ≥ 5 mm after SRP	RCT design: split-mouth Follow-up: 6 weeks NSPT delivery: full-mouth SRP, single session APC type & application: i-PRF, single Test (*n* = *10*): SRP with i-PRF Control (*n* = *10*): SRP		Values reported: Mean PPD, Mean CAL, Mean BoP, Mean PI Mean PPD reduction Mean CAL gain Mean PI reduction Timepoints: baseline and 6 weeks Other outcomes: /		Initial PT in conjunction with i-PRF proved to display significant improvement in all clinical parameters in comparison to initial PT alone.	
Amin et al. (2022) Egypt Comparison between PRP vs iPRF as an adjunct therapy in Infra-bony Pocket treatment		Setting: University (single center) *N* = *40 (Test N* = *30, Control N* = *10)* Age: Test: 34 ± 7.59, Control 35.1 ± 7.36 Gender: Test Female *n* = *14*, Male *n* = *16*; Contro*l* Female *n* = *4,* Male* n* = *6* Smoking: No Periodontal diagnosis: Stage II/III Grade B/C Site selection (*n* = n/a): single rooted teeth with bilateral interproximal defects, PPD ≥ 5 mm, interdental CAL 3 mm or > 5 mm, no tooth mobility, gingival biotype ≥ 1 mm with sufficient width of attached gingiva	RCT design: split-mouth, parallel Follow-up: 3 months NSPT delivery: full-mouth SRP, ultrasonic and manual instrumentation APC type & application: PRP/i-PRF, 3x (14 day intervals) Test 1 (*n* = *30*): SRP and PRP Test 2 (*n* = *30*): SRP and i-PRF Control (*n* = *n/a*): SRP		Values reported: Mean PPD, Mean CAL Mean PD reduction (%) Mean CAL gain (%) Timepoints: baseline, 1, 2, and 3 months Other outcomes:/		I-PRF group showed the highest reduction of PPD from baseline, while PRP group came second followed by the control group which had the lowest reduction in PPD.	
Narendran et al. (2022) India Autologous platelet-rich fibrin as an adjunct to non-surgical periodontal therapy – A follow up clinical study		Setting: University (single center) *N* = *16* Age: 35–45 years (40.56 ± 3.39) Gender: Female and Male (*n* = *n/a*) Smoking: Not reported Periodontal diagnosis: Stage III Grade n/a Site selection (*n* = *32*): single-rooted teeth with PPD ≥ 5 mm	RCT design: split-mouth Follow-up: 90 days NSPT delivery: full-mouth SRP APC type & application: PRF, single Test (*n* = *16*): SRP and PRF Control (*n* = *16*): SRP		Values reported: Mean PPD, Mean CAL Mean PPD reduction Mean CAL gain Time points: baseline, 60 and 90 days Other outcomes: /		A statistically significant improvement in PPD reduction and CAL gain when PRF was used as and adjunct to SRP in moderate periodontal pockets.	
Al-Rihaymee & Sh. Mahmood (2023) Iraq The efficacy of non-surgical platelet-rich-fibrin application on clinical periodontal parameters and periostin level in periodontitis: Clinical trial	Setting: University (single center) *N* = *14* Age: Not specified Gender: Female *n* = *2*, Male *n* = *12* Smoking: Not specified Periodontal diagnosis: Periodontitis according to 2017 World Workshop Site selection (*n* = *28*): PPD 4–6 mm, no B and L sites of multirooted teeth, teeth with endodontic lesions, grade II mobility, caries, dental prosthesis		RCT design: split-mouth Follow-up: 3 months NSPT delivery: full-mouth ultrasonic scaling (first session), manual SRP after 7 days (second session) APC type & application: PRF, single Test (*n* = *14*): SRP with saline irrigation and PRF Control (*n* = *14*): SRP with saline irrigation	Values reported: Mean PI, Mean BoP, Mean PPD, Mean RAL Mean PPD reduction Mean RAL gain Timepoints: baseline, 1 and 3 months Other outcomes: Periostin levels in GCF				Non-surgical application of PRF adjunctive to conventional SRP can significantly improve the healing process through elevated periostin levels.
Shunmuga et al. (2023) India Clinical evaluation of the combined efficacy of injectable platelet-rich fibrin along with scaling and root planing in the non-surgical periodontal therapy of stage III and grade C periodontitis patients having type 2 diabetes mellitus: a randomized controlled trial	Setting: University (single center) *N* = *23* Age: 30–75 years (51.13 ± 11.72) Gender: Female *n* = *13*, Male *n* = *10* Smoking: No Periodontal diagnosis: Stage III Grade C Other: DM2, HbA1c ≥ 7 Site selection (*n* = *46*): 2 interproximal sites with PPD ≥ 5 mm and attachment loss in each mandibular posterior quadrant. No teeth with pseudo pockets/advanced periodontal destruction, mobility, and grade IV FI		RCT design: split-mouth Follow-up: 6 months NSPT delivery: full-mouth SRP, ultrasonic and manual instrumentation APC type & application: i-PRF, single Test (*n* = *23*): SRP with saline irrigation and i-PRF Control (*n* = *23*): SRP with saline irrigatiom	Values reported: Mean PI, Mean MGI, Mean PPD, Mean CAL, Mean BoP, Mean GR Mean PI change Mean mGI change Mean PPD reduction Mean CAL gain Mean BoP change Mean GR increase Timepoints: baseline, 3 and 6 months Other outcomes: HbA1c levels at baseline and 6 months (change not statistically significant)				The use of i-PRF adjunctive to SRP did not provide any additional advantages over SRP with saline in stage III grade C periodontitis DM2 subjects.
Cin et al. (2024) Turkey Efficacy of injectable platelet‐rich fibrin on clinical and biochemical parameters in non‐surgical periodontal treatment: a split‐mouth randomized controlled trial	Setting: University (single center) *N* = *17* Age: 37.41 ± 5.84 years Gender: Female *n* = *10*, Male *n* = *7* Smoking: No Periodontal diagnosis: Stage III Grade B Site selection (*n* = *34)*: contralateral sites with interproximal PPD > 6 mm, no teeth with periapical lesions, FI, and ≥ degree 1 mobility		RCT design: split-mouth Follow-up: 6 months NSPT delivery: full-mouth supragingival scaling (first session), SRP by manual instrumentation after 7 days (second session) APC type & application: i-PRF, single Test (*n* = *17*): SRP with saline irrigation and i-PRF Control (*n* = *17*): SRP with saline irrigation	Values reported: Mean GI, Mean BoP, Mean PI, Mean PPD, Mean CAL, Mean GR, Mean PPD reduction Mean CAL gain Mean GR increase Timepoints: baseline, 1, 3 and 6 months Other outcomes: GCF levels of VEFG, TNF‐α, and IL‐10 at baseline, 3rd, 7th, and 14th day				Subgingival i-PRF injection adjunct to conventional SRP in deep periodontal pockets may improve clinical periodontal outcomes without causing any morbidity in patients. Administration of i-PRF increased the VEGF and IL-10 levels and decreased TNF-α levels in GCF.

Abbreviations: *RCT *randomized controlled trial; *NSPT *non-surgical periodontal therapy; *CP *Chronic Periodontitis; *SRP *scaling and root planing; *APC *autologous platelet concentrate; *PRP *platelet-rich plasma; *PRF *platelet-rich fibrin; *i-PRF *injectable platelet-rich fibrin; *PRGF *plasma rich in growth factors; *HA *hyaluronic acid; *CAL *clinical attachment level;* RAL *relative attachment level; *PPD *probing pocket depth; *BoP *bleeding on probing; *PI *plaque index; *GR *gingival recession; *MGI *modified gingival index; *SBI *sulcus bleeding index; *FI *furcation involvement; *GCF *gingival crevicular fluid; *Col-1 *collagen-1;* TGF- β *transforming growth factor β; *TNF‐α *tumor necrosis factor α; *VEGF *vascular endothelial growth factor; *IL-10 *interleukin-10; *DM2 *Type 2 Diabetes Mellitus;* HbA1c *glycated hemoglobin;* B *buccal; *L *lingual

**Table 2:** Summary of APC preparation protocols, delivery, and post-operative instructions

Study	APC	Preparation	Application	Instructions
Agarwal & Dev Gupta (2015)	PRP	Protocol: [48] Sample: 60 ml of whole blood mixed with 7 ml of citrate anticoagulant solution Centrifugation: 2 cycles: 2400 rpm for 10 min and 3600 rpm for 15 min APC: 7 ml of PRP with 1 ml of 10% calcium chloride solution mixed with 1,000 U.S. units of topical thrombin before application	Site preparation: SRP Delivery: Blunt 22G needle placed at the bottom of the pocket until the pocket was overfilled Delay: No information Stabilization: Pressure with a moist gauze for 5 min	Refrain from tooth brushing around the treated sites for 2 days. A 0.12% CHX mouthwash to be used twice daily for 2 weeks
Amin et al. (2022)	PRP	Protocol: No information Sample: 2 Acid citric dextrose 8.5 ml tubes of whole blood Centrifugation: 2000 rpm for 7 min at room temperature (Heraeus Mega-fuge™ 16R centrifuge, Thermo scientific™, USA) APC: Top layer after centrifugation, collected by a plastic syringe	Site preparation: SRP, cleansed and anaesthetized with lidocaine topical gel Delivery: Injection into the gingival sulcus with a microneedle until the blanching and fullness of gingiva was noted Delay: No information Stabilization: Pressure with a moist gauze for 5 min *Repeated at day 1, 14, and 28	Regular oral hygiene and CHX mouthwash rinse twice daily for a week
Özcan et al. (2021)	PRF	Protocol: [23] Sample: 2 10 ml tubes of whole blood without anticoagulant Centrifugation: 3000 rpm (400 g) for 10 min (Nuve, CN 180, Bench-Top Centrifuge, Ankara, Turkey) APC: Fibrin clot in the centre of the tube,compressed by tongue depressors to mm thickness, cut into 3 × 5 mm dimensions	Site preparation: SRP Delivery: Insertion into the pockets from the base up to the gingival margin Delay: No information Stabilization: No information	Resume tooth brushing the next day, no antibiotic medication or antimicrobial mouthwash prescribed
Narendran et al. (2022)	PRF	Protocol: [49] Sample: Whole blood without anticoagulants. Centrifugation: 2700 rpm for 12 min (Remi R-8c BL centrifugation system) APC: Topmost layer, picked up by forcps and separated using scissors, compressed using Osung PRF/GRF Box, and trimmed	Site preparation: SRP Delivery: Insertion with the help of gingival retraction cord packer Delay: No information Stabilization: Digital pressure through west gauze for 5–10 min, chair rest for 30–45 min, periodontal dressing (GC Coe Pak) placed on both sites	No information
Al-Rihaymee & Sh. Mahmood (2023)	PRF	Protocol: [23] Sample: 10 ml of whole blood in a sterile glass tube without anticoagulants Centrifugation: 3000 rpm for 10 min APC: Fibrin clot in the centre of the tube, separated by tweezers and surgical scissors, gently compressed for 10 min to 1 mm thickness	Site preparation: SRP Delivery: Insertion into the base of the pocket, filling up to the marginal gingiva Delay: No information Stabilization: No information	Resume tooth brushing the next day, no mouthwash advised following the treatment
Panda et al. (2020)	PRGF	Protocol: [50] Sample: 9 ml of whole blood in vacutainers containing 10% trisodium citrate Centrifugation: 580 g for 8 min at room temperature (BTI-Endoret System IV Centrifuge, Biotechnology Institute, Vitoria, Spain) APC: middle of the test tube, collected by a pipette, activated with 0.2 ml 10% Calcium Chloride to obtain PRGF gel	Site preparation: SRP Delivery: Pistoned into the pockets through a blunt cannula Delay: 5–10 min after preparation Stabilization: No information	Oral hygiene instructions, no antibiotics or anti-microbial agents prescribed
Vučković et al. (2020)	i-PRF	Protocol: No information Sample: 2 10 ml tubes of whole blood without anticoagulant Centrifugation: 700 rpm (60 g) for 3 min at room temperature by a Duo Centrifuge (Process for PRF, Nice, France) APC: Upper liquid layer taken by a syringe	Site preparation: SRP Delivery: Through perforatioons at the point of interdental space on individually formed occlusal splints Delay: No information Stabilization: Splint, removal after 15 min	No information
Albonni et al. (2021)	i-PRF	Protocol: [51] Sample: 20 ml of whole blood in 2 sterile plastic tubes without anticoagulant Centrifugation: 700 rpm (60 g) for 3 min; Duo-centrifuge (Process for PRF) APC: 1 ml of upper liquid layer collected per tube in an insulin syringe connected to a 29G needle (Insu/Light 1 ml)	Site preparation: SRP under local anasthesia Delivery: By a blunt 29G needle placed at the bottom of the pocket until the pocket was overfilled Delay: Less than 15 min Stabilization: No information	Resume tooth brushing around the treated sites and rinse twice daily with 0.12% CHX for 2 weeks, starting the following day
Rakhewar et al. (2021)	i-PRF	Protocol: No information Sample: 2 10 ml tubes of whole blood without anticoagulant Centrifugation: 700 rpm for 3 min (60 g) at room temperature (Remi R-8C Laboratory Centrifuge processer) APC: Upper liquid layer taken as i-PRF	Site preparation: SRP Delivery: Injection of i-PRF into periodontal pockets Delay: No information Stabilization: No information	No information
Amin et al. (2022)	i-PRF	Protocol: [52] Sample: 2 10 ml tubes of whole blood without anticoagulant; Centrifugation: 700 rpm for 3 min at room temperature (Heraeus Mega-fuge™ 16R centrifuge, Thermo scientific™, USA) APC: Upper liquid layer taken as i-PRF	Site preparation: SRP, cleansed and anaesthetized with Lidocaine topical gel Delivery: Injection into the gingival sulcus with a microneedle until the blanching and fullness of gingiva was noted Delay: No information Stabilization: Pressure with a moist gauze on the site for 5 min following delivery *Repeated at day 1, 14, and 28	Regular oral hygiene and mouthwash with CHX twice a day for a week
Shunmuga et al. (2023)	i-PRF	Protocol: [53, 54] Sample: 10 ml of whole blood in sterile non-coated plastic vacutainers without anticoagulant Centrifugation: 700 rpm for 4 min (Labtech-Dentifuge) APC: Top layer of yellow liquid retrieved using a disposable syringe	Site preparation: SRP Delivery: Subgingival, using a syringe with a blunt cannula needle in an apico-coronal direction until the pocket entrance was reached Delay: Less that 15 min, Stabilization: Resorbable periodontal dressing (Reso-Pack)	Refrain from tooth brushing around the treated sites for 2 days, advised to use 1.12% CHX mouthwash twice daily for 2 weeks
Cin et al. (2024)	i-PRF	Protocol: [51] Sample: 10 ml of whole blood in a plastic tube without anticoagulant Centrifugation: 700 rpm (60 g) for 3 min (Duo-centrifuge (PRF Process, Nice, France) APC: Upper layer collected and placed into a 2.5-cc dental injector	Site preparation: SRP under local anasthesia Delivery: Inner epithelial layer of the pocket, from the bottom of the pocket moving coronally, targeting the midpoint of the sulcus epithelium. The remaining i-PRF was injected into the gingival sulcus with a 30G irrigation needle until the pocket was overfilled Delay: No information Stabilization: Saliva isolation for 15 min, compression through sterile wet gauze	Avoid using the interproximal brush or flossing for the first day. No antimicrobial medication prescribed

Abbreviations: *SRP *scaling and root planing; *APC *autologous platelet concentrates; *PRP *platelet-rich plasma; *PRF *platelet-rich fibrin;* i-PRF *injectable platelet-rich fibrin; *PRGF *plasma rich in growth factors;* CHX *chlorhexidine

The majority of the included studies had a split-mouth design, while the study by Amin et al. employed a combination of split-mouth and parallel designs. All the studies were conducted within single-centre university settings, most in Middle Eastern and South Asian regions. The publication year was similar in all studies (2020–2024) except for Agarwal & Dev Gupta (2015), corresponding with PRP as an early subtype of APCs used in the given study. Studies varied significantly in site selection criteria, follow-up times, post-operative instructions, and handling of possible modifying factors, such as smoking. All included studies, except for Shunmuga et al., included systemically healthy subjects, while the latter included subjects with uncontrolled (HbA1c values > 7) Type 2 Diabetes Mellitus (DM2) and was therefore excluded from quantitative synthesis.

The included studies scarcely detailed the NSPT delivery protocols. Most employed a combination of hand and powered instruments, with full-mouth SRP conducted either in a single session or two sessions over a 24-h period. Al-Rihaymee et al. and Cin et al. reported NSPT delivery in two sessions, with subgingival instrumentation following supragingival scaling after 7 days. The majority of the studies employed a single application of APCs; however, Amin et al. administered PRP and i-PRF three times at 14-day intervals. Furthermore, due to the different preparation methods of different APC subtypes, their physical state differed, with eight studies utilizing liquid APC forms (PRP, iPRF, PRGF) and three studies using a solid APC form (PRF membrane).

Four studies [30–33] investigated biomarker changes associated with the adjunctive use of APCs to NSPT; however, the outcomes were insufficiently similar for quantitative analysis. Furthermore, two studies [43, 44] reported on pocket closure; however, the low number of studies and different methodologies and definitions used did not allow for a quantitative synthesis. None of the included studies reported the occurrence of radiographic changes or PROMs.

### Synthesis

Among the eleven included studies, the distribution of investigations into adjunctive APC applications was as follows: PRP was examined in two studies [45, 46], PRF in three studies [32, 33, 35], i-PRF in five studies [30, 31, 34, 46, 47], and PRGF was investigated in one study [43].

**Fig. 2:**
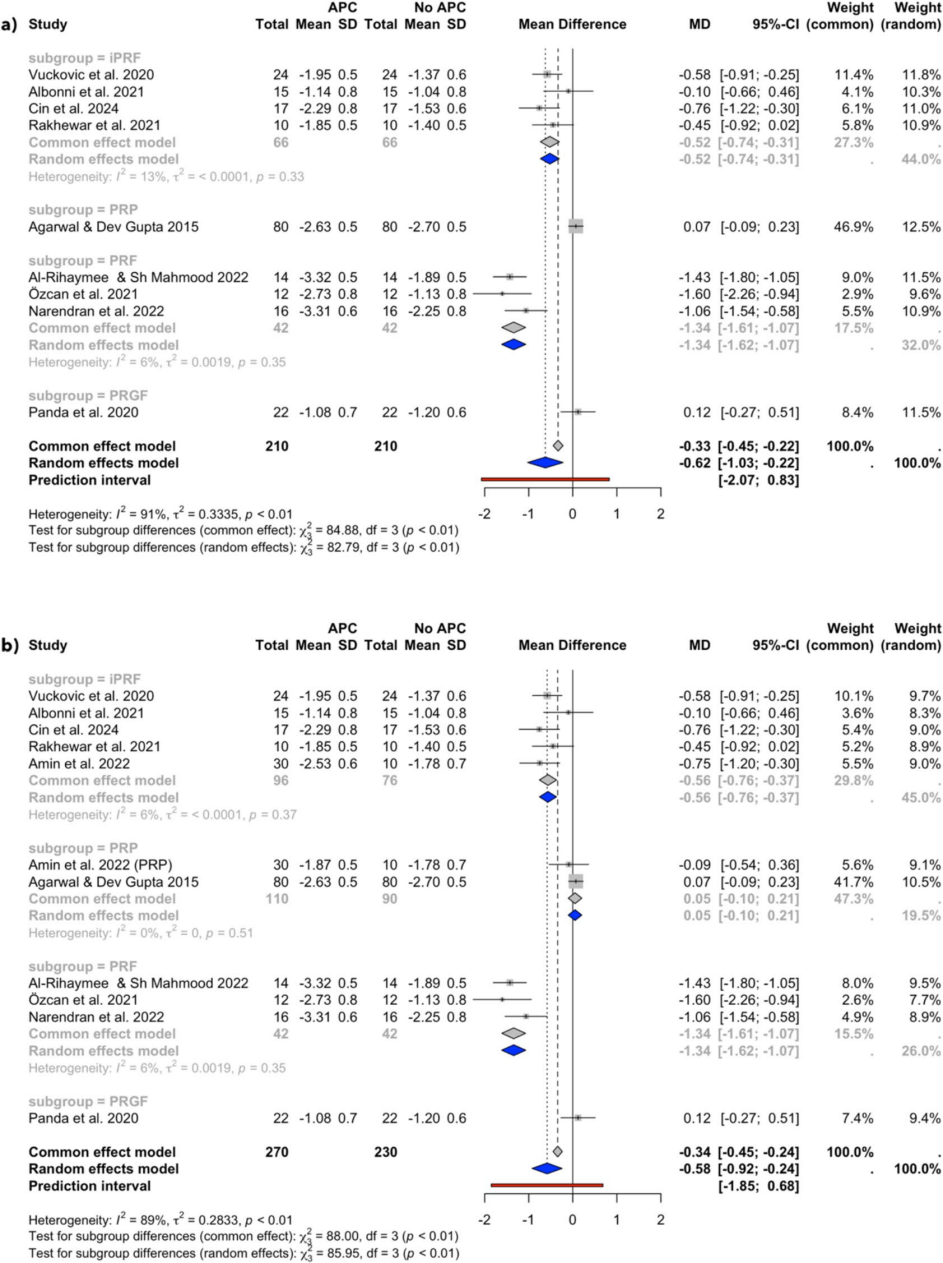
PPD changes at the 6–12 week follow-up in (**a**) studies with a single APC application and (**b**) all included studies

**Fig. 3:**
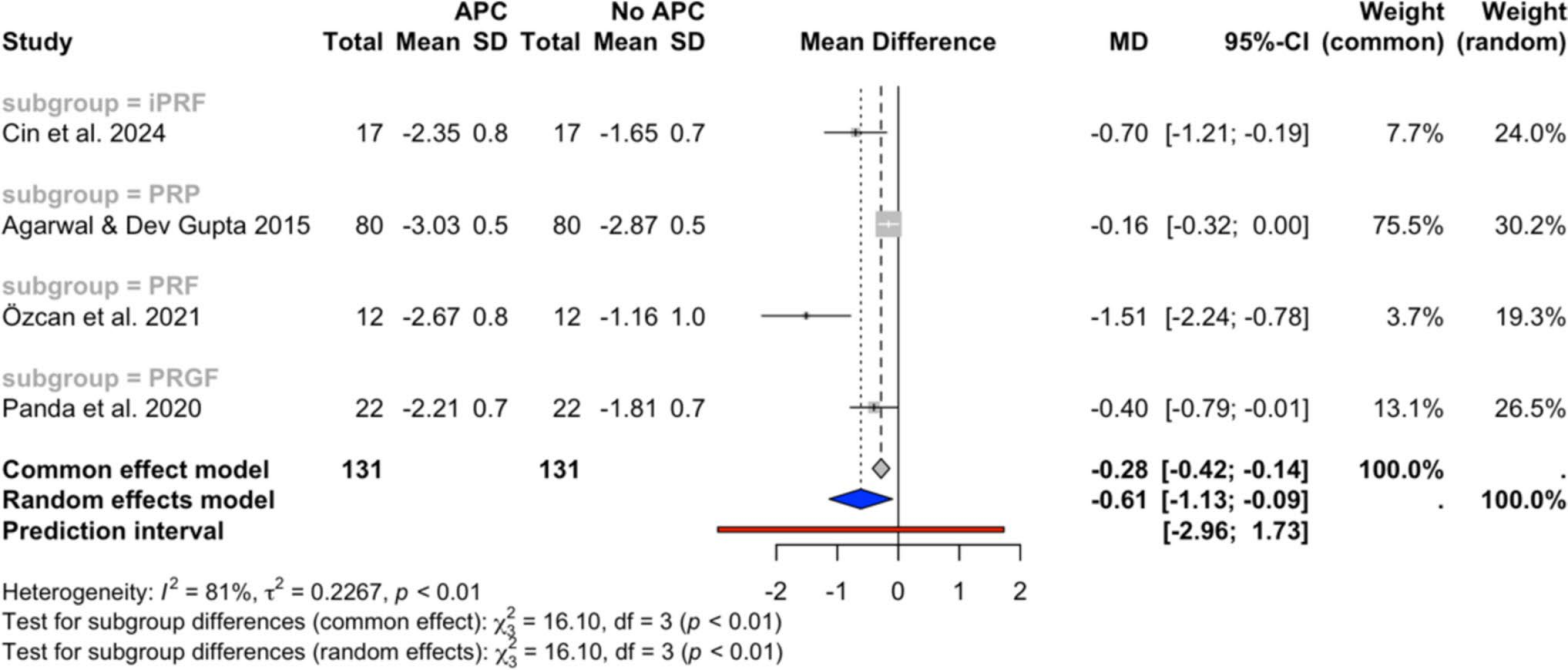
PPD changes at the 6-month follow-up in studies with a single APC application

**Fig. 4:**
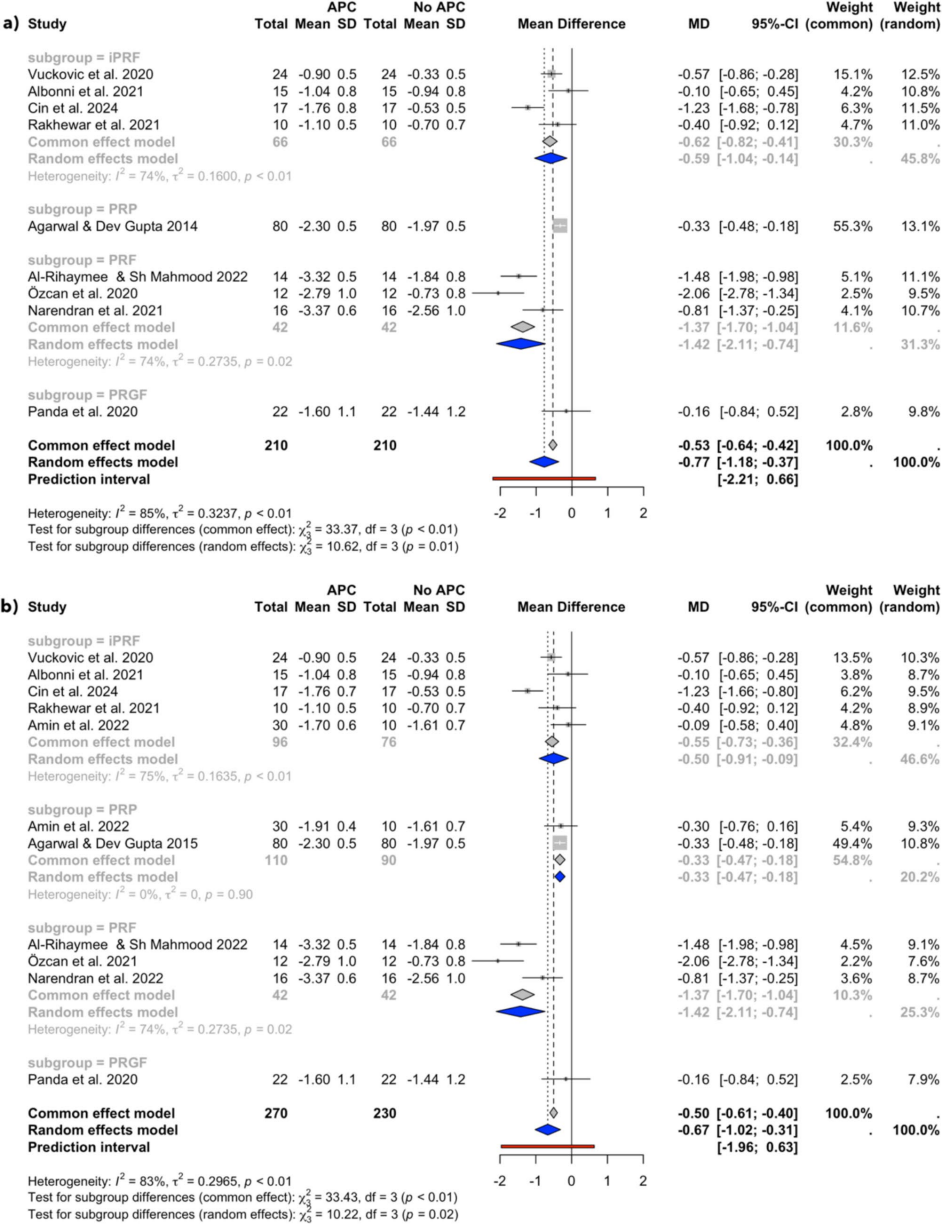
CAL changes at the 6–12 week follow-up in (**a**) studies with a single APC application and (**b**) all included studies

**Fig. 5:**
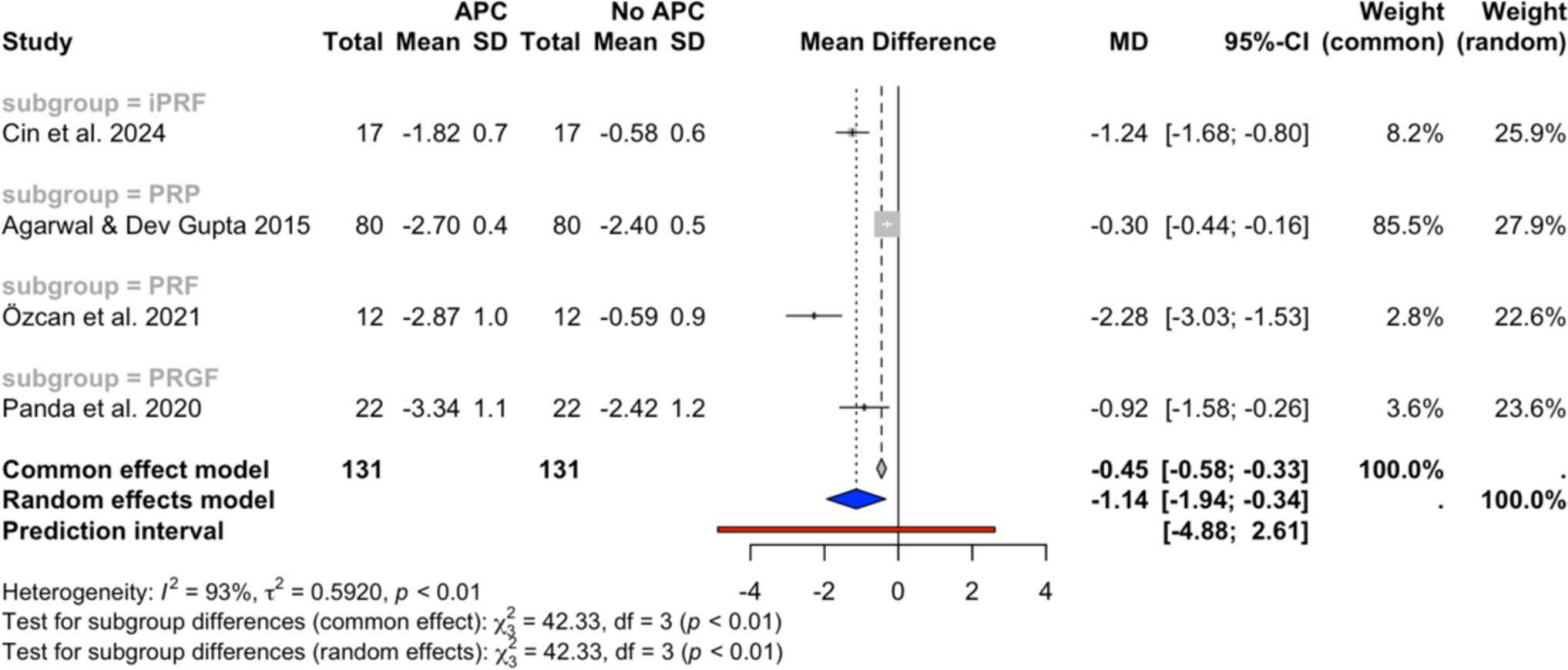
CAL changes at the 6-month follow-up

**Fig. 6:**
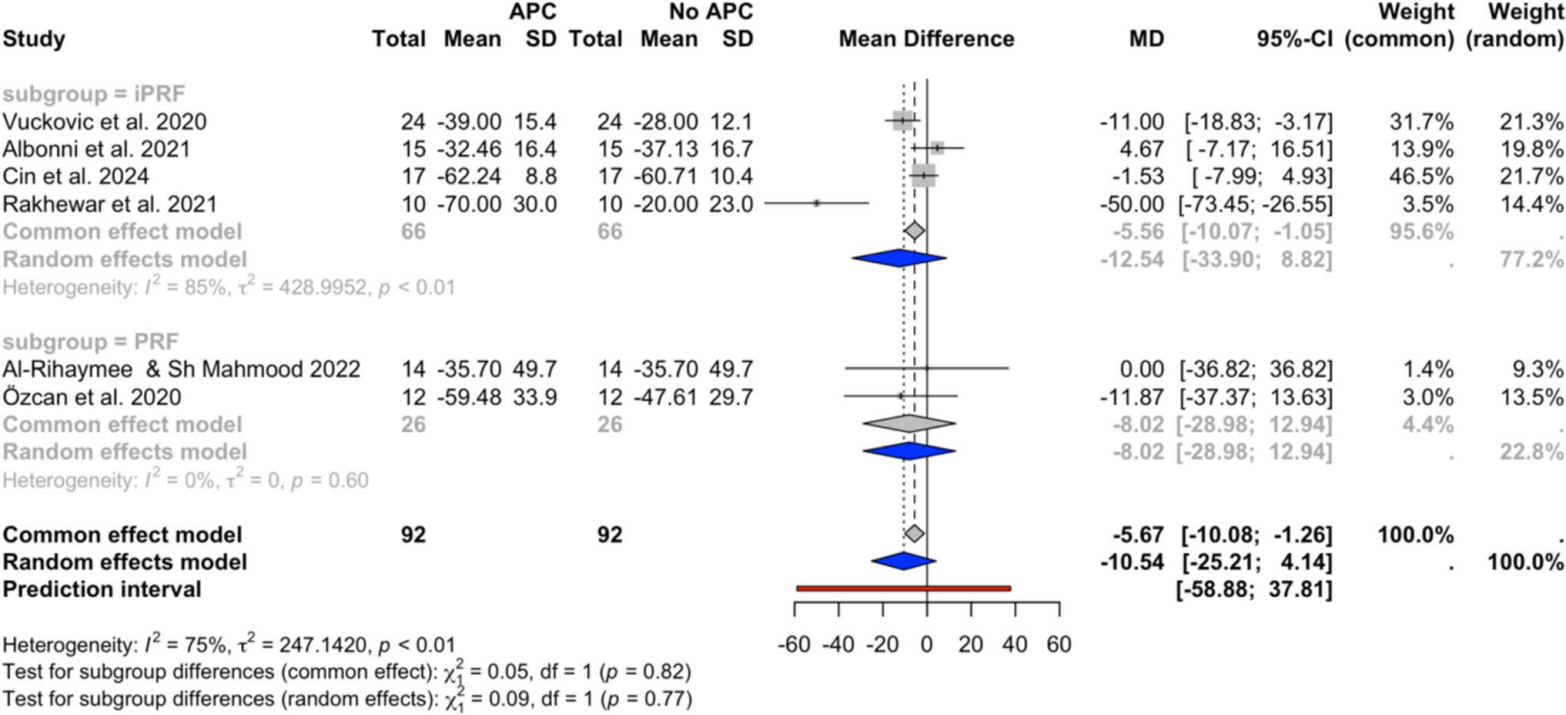
BoP changes at the 6–12 week follow-up

Quantitative synthesis was performed on 11 study groups from 10 independent studies, divided into 4 subgroups by the type of APCs used (PRP, PRF, i-PRF, and PRGF). Meta-analyses were possible for the following outcomes: PPD reduction and CAL gain at 6–12 week and 6-month follow-ups, and BoP reduction at 6–12 weeks follow-up. The meta-analysis results are illustrated in Figs. 2, 3, 4, 5, and 6. The sensitivity analysis results, which excluded the high risk of bias studies [34, 46], are illustrated in Figs. 9, 10, and 11 (Appendix 3) and presented in Table 3.

**Table 3:** Sensitivity analysis comparing the main single APC application results at the 6–12 week follow-up with high-risk studies excluded

Outcome measure	Analysis type	Effect size (MD)	95% CI	*P*-value	Number of studies	Heterogeneity (I^2^)
PPD reduction (mm)	Main	− 0.62	− 1.03; − 0.22	< 0.01	9	91%
	Sensitivity	− 0.65	− 1.10; − 0.19	< 0.01	8	92%
CAL gain (mm)	Main	− 0.77	− 1.18; − 0.37	< 0.01	9	85%
	Sensitivity	− 0.82	− 1.27; − 0.36	< 0.01	8	87%
BoP reduction (%)	Main	− 10.54	− 25.21; 4.14	< 0.01	6	75%
	Sensitivity	− 3.88	− 11.04; 3.29	0.19	5	34%

### PPD reduction

Figure 2a illustrates the results of the meta-analysis of PPD reduction conducted across 4 subgroups (iPRF, PRP, PRF, and PRGF) from 9 studies with a single APC application [30, 32–35, 43–45, 47] at 6–12 weeks follow-up. The overall random effects model showed a pooled MD of −0.62 mm (95% CI: −1.03, −0.22, *I*^*2*^ = 91%), indicating a statistically significant reduction of PPD in the APC group compared to the no APC group. Similarly, after the inclusion of the study conducted by Amin et al., which employed multiple APC applications, the overall random-effects model displayed a pooled MD of −0.58 mm (95% CI: −0.92, −0.24, *I*^*2*^ = 89%), also favouring the APC group.

Figure 3 presents the meta-analysis outcomes at the 6-month timepoint for 4 subgroups (iPRF, PRP, PRF, and PRGF) aggregated from 4 studies [30, 32, 43, 45]. The overall random effects model demonstrated a statistically significant reduction in PPD with the MD of −0.61 (95% CI: −1.13, −0.09, *I*^*2*^ = 81%), favouring the APC group.

### CAL gain

Figure 4a displays the results of the meta-analysis for CAL at the 6–12 weeks follow-up for studies with a single APC application, including 4 subgroups (iPRF, PRP, PRF, and PRGF) across 8 studies [30, 32–35, 44, 45, 47]. The random effects model showed a statistically significant improvement in CAL with an MD of −0.77 (95% CI: −1.18 to −0.37, *I*^*2*^ = 85%), favouring the APC group. Similarly, the analysis presented in Fig. 4b, which included the study by Amin et al., also favoured the APC group, with an MD of −0.67 mm (95% CI: −1.02 to −0.31, *I*^*2*^ = 83%).

The results of the meta-analysis of CAL changes at the 6-month follow-up are displayed in Fig. 5. It included 4 subgroups (iPRF, PRP, PRF, and PRGF) across 4 studies, of which all utilized a single APC application [30, 32, 43, 45]. The overall random effect model showed a statistically significant improvement in CAL favouring the APC group, with an MD of −1.14 (95% CI: −1.94 to −0.34, *I*^*2*^ = 93%).

### BoP change

The results of the meta-analysis of BoP percentage changes at the 6–12 weeks follow-up are displayed in Fig. 6. The analysis included 2 subgroups (iPRF and PRF) across 6 studies with a single APC application [30, 32–34, 44, 47]. The overall random effects model did not display a statistically significant result, with an MD of −10.54 (95% CI: −25.21 to 4.14, *I*^*2*^ = 75%).

### Heterogeneity

The overall analysis consistently displayed high heterogeneity (*I*^*2*^ values ranging from 75 to 91%), while the subgroup analyses revealed varying levels of heterogeneity. Low heterogeneity (*I*^*2*^ values ranging from 0 to 6%) was present in the 6–12 weeks follow-up PPD change analyses (Fig. 2) and the PRP subgroup of the 6–12 weeks follow-up CAL change analysis (Fig. 4), while high heterogeneity (*I*^*2*^ values ranging from 74 to 75%) was revealed in the 6–12 weeks follow-up CAL change iPRF and PRF subgroup analyses (Fig. 4).

### Risk of bias

**Fig. 7:**
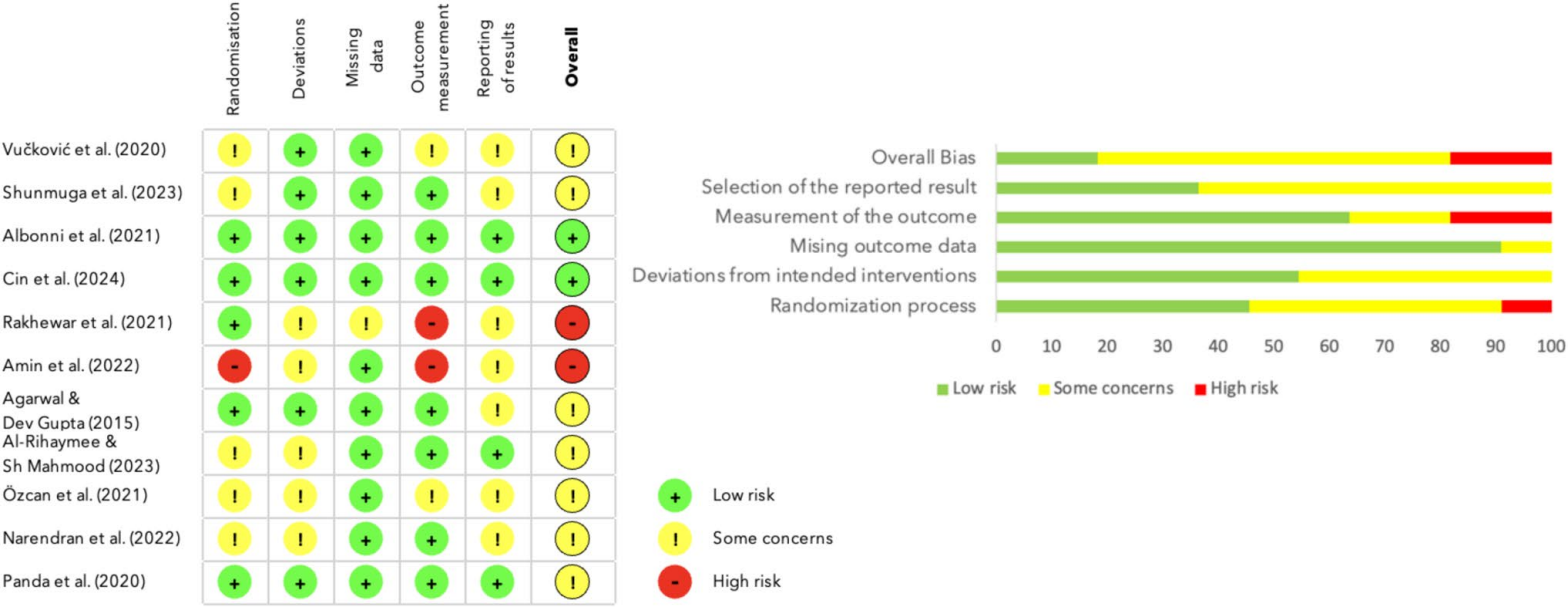
Risk of bias presented for individual studies and as a percentage of included studies according to domain

The results from the RoB 2.0 tool are presented in Fig. 7. The risk of bias assessment revealed different levels of methodological quality among the included studies. Two studies (18.2%) were rated as having a low risk of bias across all domains. Seven studies (63.6%) exhibited an unclear risk of bias, predominantly due to insufficient details regarding random sequence generation and allocation concealment. The remaining two studies (18.2%) were judged to have a high risk of bias, mainly attributed to the lack of blinding of participants and outcome assessors.

**Fig. 8:**
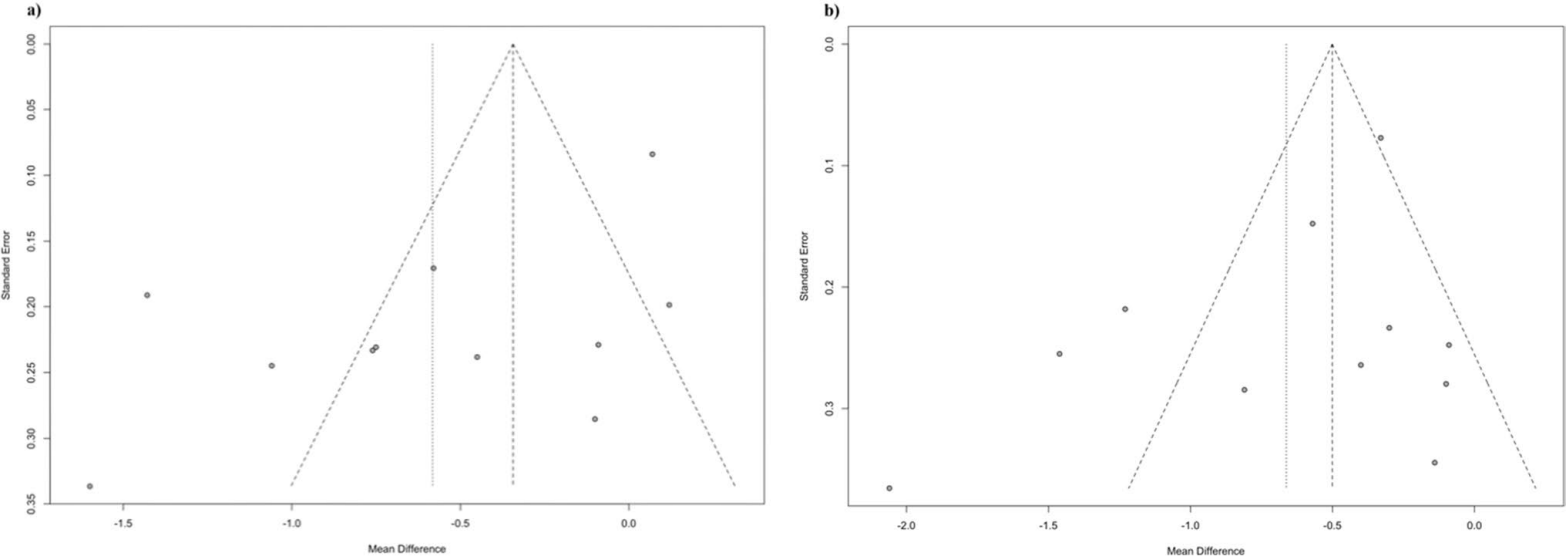
Funnel plots assessing publication bias for a) PPD changes and b) CAL changes at the 6–12 week follow-up in all included studies

Figure 8 presents funnel plots assessing publication bias in PPD and CAL changes meta-analyses at the 6–12 weeks follow-up in all included studies. Moderate asymmetry was observed in both. Comparatively, greater asymmetry was observed for the PPD rather than CAL meta-analysis.

### Certainty of evidence

The strength of evidence was deemed severely limited due to the very low degree of certainty assessed across all examined outcomes. The GRADE assessment results are presented in Table 4, with detailed explanations in the footnotes.

**Table 4:**
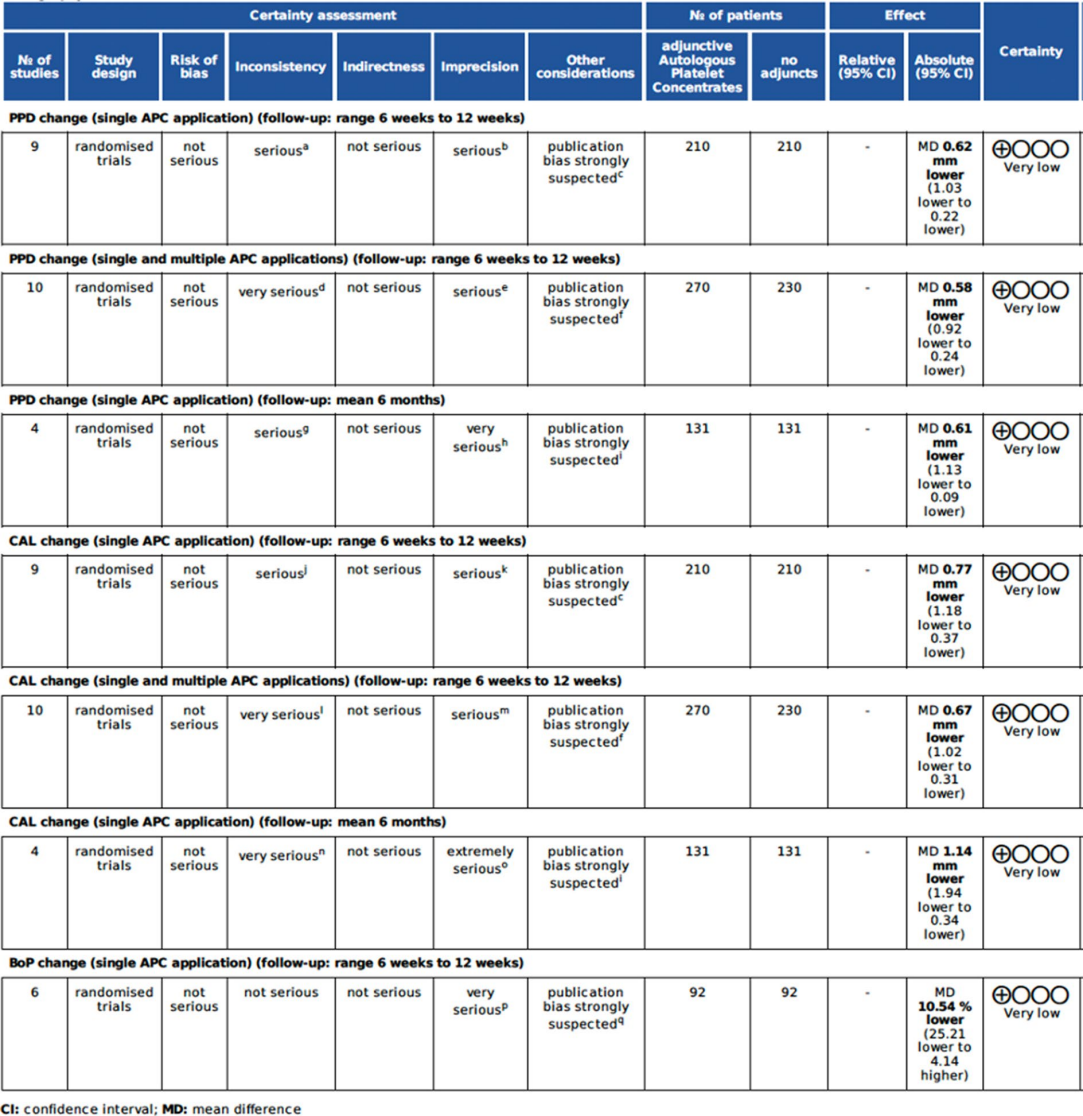
GRADE assessment

Abbreviations:* CI *Confidence interval; *MD* Mean difference

a. *I*^*2*^ = 91%, minimal overlap of confidence intervals, observed differences across subgroup effects.

b. Wide confidence interval (98% CI: −1,18; −0.37), wide prediction interval (PI: −2.01; 0.83), *I*^*2*^ = 91%, small sample size.

c. Unavailability of protocols or registrations in 6 out of 9 studies, funnel plot inspection (Fig. 8).

d. *I*^*2*^ = 89%, minimal overlap of confidence intervals, observed differences across subgroup effects, studies with single and multiple APC applications.

e. Wide confidence interval (98% CI: −1,85; −0.68), wide prediction interval (PI: −1.85; 0.68), *I*^*2*^ = 81%, small sample size.

f. Unavailability of protocols or registrations in 7 out of 10 studies.

g. *I*^*2*^ = 81%, minimal overlap of confidence intervals, observed differences across subgroup effects.

h. Wide confidence interval (98% CI: −1,13; −0.09), wide prediction interval (PI: −2.96; 1.73), *I*^*2*^ = 81%, small sample size.

i. Unavailability of protocols or registrations in 3 out of 4 studies.

j. *I*^*2*^ = 85%, minimal overlap of confidence intervals, observed differences across subgroup effects.

k. Wide confidence interval (98% CI: −1,18; −0.37), wide prediction interval (PI: −2.21; 0.66), *I*^*2*^ = 85%, small sample size.

l. *I*^*2*^ = 83%, observed differences across subgroup effects, studies with single and multiple APC applications.

m. Wide confidence interval (98% CI: −1,02; −0.31), prediction interval (PI: −1.96; 0.63), *I*^*2*^ = 83%, small sample size.

n. *I*^*2*^ = 93%, observed differences across subgroup effects, minimal overlap of the confidence intervals, and the inclusion of studies with single and multiple APC applications.

o. The confidence interval (98% CI: −1.94; −0.34), wide overall prediction interval (PI: −4.88; 2.61), and the high I^2^ value of 93%, together with a small sample size, suggest extremely serious imprecision.

p. The confidence interval (98% CI: −25.21; 4.14), wide prediction interval (PI: −58.88; 37.81), and the I^2^ value of 75%, together with a small sample size, suggest extremely serious imprecision.

q. Unavailability of protocols or registrations in 3 out of 6 studies.

## Discussion

This systematic review investigated the potential benefits of using different APC subtypes and APCs as a whole as an adjunctive treatment modality in NSPT. Despite the substantial variations in study populations, protocols, number of applications, and the type of APCs used, their use appears to benefit periodontal treatment outcomes.

The meta-analyses of single-application studies favoured the APCs group, indicating a greater reduction in PPD of 0.62 mm (95% CI: −1.03, −0.22) and further gain in CAL of 0.77 mm (95% CI: −1.18, −0.37) at 6–12 weeks follow-up, as well as a greater reduction in PPD of 0.61 mm (95% CI: −1.13, −0.09) and further gain in CAL of 1.14 mm (95% CI: −1.94, −0.34) at the 6-month follow-up compared to NSPT group alone. The difference in BoP reduction between the groups at the 6–12 week follow-up was not statistically significant (MD = −10.54; 95% CI: −25.21, 4.14). The inclusion of the study by Amin et al., which applied APCs multiple times during the trial period, did not significantly influence the overall results of the meta-analyses. The analyses nevertheless favoured the APCs group, indicating a higher PPD reduction and CAL gain at 6–12 weeks follow-up (MD = −0.58 mm; 95% CI: −0.92, −0.24, and MD = −0.67 mm; 95% CI: −1.02, −0.31, respectively), and at the 6-month follow-up (MD = −0.61; 95% CI: −1.13, −0.09, and MD = −1.14; 95% CI: −1.94, −0.34, respectively) when compared to NSPT alone. Similarly, the exclusion of the studies by Amin et al. and Rakhewar et al., which were deemed at high risk of bias, did not significantly affect the results (Appendix 3). The results at the 3-month follow-up still favoured the APC group in PPD reduction (MD =—0.65; 95% CI: −1.10, −0.19) and CAL gain (MD = −0.82, 95% CI: −1.27, −0.36), while no statistically significant difference was detected in BoP reduction between the groups (MD = −3.88, 95% CI: −11–04, 3.29).

We observed varying degrees of mean PPD reduction across the included studies. To assess the included studies’ treatment efficacy, we compared the mean PPD reduction of the control groups who underwent NSPT without adjuncts to the benchmark described by Suvan et al., who showed a mean PPD reduction of 1.5 mm and 1.6 mm in initially shallow pockets (4–6 mm) respectively at 3–4 months and 6–9 months, and 2.6 mm at both timepoints for initially deep pockets (> 6 mm) [1]. The results of studies reporting a mean PPD < 7 mm were consistent with Suvan et al.’s findings at both 6–12 weeks (ranging from −1.04 ± 0.8 mm to −2.25 ± 0.8 mm) and 6-month follow-ups (ranging from −1.16 ± 1 mm to −2.87 ± 0.5 mm) [32–35, 43–47]. However, Cin et al. reported lower than expected PPD reductions for deep pockets at both 3 and 6-month follow-ups (−1.53 ± 0.6 mm and −1.65 ± 0.7 mm, respectively) [30].

The magnitude of the additional PPD reduction 6 months after treatment (0.61 mm; 95% CI: −1.13, −0.09), even though not measurable with a conventional periodontal probe in a single site, is considered clinically relevant in the periodontal literature, when it is obtained as mean. Our findings align with the findings of a systematic review published by Teughels et al., investigating the efficacy of adjunctive systemic antimicrobial use in NSPT, which unquestionably leads to further pocket reductions, although with potential adverse events [55]. They reported an additional PPD reduction of 0.417 mm (95% CI: 0.306; 0.528) for initially shallow and 0.969 mm (95% CI: 0.755; 1.183) for initially deep pockets at 6 months post-treatment. Furthermore, they reported 0.389 mm (95% CI: 0.267; 0.511) of additional CAL gain 6 months post-treatment. The CAL gain in the present meta-analysis was higher at 1.14 mm (95% CI: −1.94; −0.34); however, with a wide prediction interval (PI: −4.88; 2.61), it is less reliable.

Only two of the included studies reported on pocket closure, which prevented us from conducting a meta-analysis. Panda et al. reported solely the change in the number of pockets with PPD > 4 mm and PPD < 4 mm, which was found to statistically significantly favour the PRGF group at 6 months (90.9% of the sites reduced to PPD < 4 mm in the test group as compared to 59.1% in the control group) [43]. Albonni et al. defined pocked closure as PPD = 4 mm without BoP and reported statistically insignificant results of 14.5% pocket closure in the i-PRF group and 12.4% pocket closure in the control group [44]. A comparison with previously published literature did not seem appropriate because of the difference in definitions of ’pocket closure’.

The use of the first APC generation as an enhancement factor for wound healing in oral and maxillofacial surgery, PRP, was first described by Whitman et al. in 1997 [12]. The preparation method, without a standardized protocol, included the addition of xenogeneic thrombin and calcium chloride. Similarly, sodium citrate is added as an anticoagulant during the preparation of PRGF [56], which raised concerns about potential adverse effects due to the addition of exogenous substances. Subsequently, the second generation of APCs was introduced to simplify blood processing without biochemical modifications, making them fully autologous products formed through natural polymerization during centrifugation and significantly reducing the risk of adverse reactions compared to synthetic materials. [13, 14, 29, 49, 52, 57, 58].

APCs mimic the end stage of the coagulation cascade by forming a fibrin matrix. Its structural properties depend on the centrifugation speed, with lower centrifugation speeds creating a denser fibrin network. The fibrin matrix enables a slow, controlled release of GFs as it undergoes degradation, ensuring a prolonged availability of GFs at the injury site and allowing a sustained effect on tissue healing and regeneration [16, 59, 60]. Platelets’ α-granules contain storage pools of GFs, specifically basic fibroblast growth factor (bFGF), vascular endothelial growth factor (VEGF), insulin-like growth factor-1 (IGF-1), transforming growth factor β−1 (TGF-β1), and platelet-derived growth factor-BB (PDGF-BB) [18]. Furthermore, they release cytokines, including platelet factor 4 (PF4) and CD40 ligand (CD40L), chemokines, and newly synthesized active metabolites [20]. Although, theoretically, substantial differences exist in the production of GFs among the three APC preparation methods (PRP, PRF, and CGF), only the levels of bFGF in CGF and PRF have been demonstrated to be significantly higher than those in activated PRP [15]. However, Kobayashi et al. demonstrated that APCs exhibit different release kinetics. While PRP exhibited the advantage of releasing substantially higher protein levels at earlier time points, PRF displayed a consistent and sustained release of growth factors over a 10-day duration [61]. The release of GFs from platelets stimulates cell recruitment, proliferation, differentiation, angiogenesis, and extracellular matrix synthesis, which serves as a scaffold for cell migration and offers structural support throughout the healing process [15–20].

Furthermore, APCs can modulate the immune response by downregulating the expression of proinflammatory cytokines, such as interleukin (IL) 1 beta (IL-1β), IL-6, and tumour necrosis factor-alpha (TNF-α), thereby reducing inflammation and promoting healing [62]. They also possess anti-inflammatory properties that help modulate the immune response at the treatment site, promoting tissue remodelling and maturation [17, 21, 22, 63]. Three of the studies included in the present systematic review reported on biomarker-level changes in gingival crevicular fluid (GCF) after APC applications. Cin et al. found that the administration of i-PRF increased the levels of VEGF and IL-10 while decreasing TNF-α levels in GCF [30]. Özcan et al. observed an increased collagen-1 (Col-1) and transforming growth factor-beta (TGF-β) expression [32], and Al-Rihaymee & Mahmoud observed elevated GCF periostin levels following the application of PRF [33].

Therefore, the ability of APCs to provide structural support and stimulate cell growth, differentiation, and angiogenesis, alongside their immunomodulating and anti-inflammatory properties, can significantly contribute to enhanced healing. Specifically, the higher PPD reduction and CAL gain observed in the APCs group underscore their efficacy in promoting rapid tissue regeneration and improving periodontal clinical outcomes.

None of the included studies reported harmful or adverse events following the APC application. The only discomfort experienced was due to repeated blood collection after failing to find an appropriate blood vessel [47], suggesting utilizing APCs is a clinically safe procedure. However, their use in clinical practice involves several considerations, including costs, time consumption, and potential logistical challenges. APC preparation can be costly due to the need for specialized equipment for sample processing and trained personnel for sample collection. Their preparation and application may require longer appointment times, which adds another layer of complexity for the practitioners considering this treatment modality.

The meta-analyses indicated substantial overall heterogeneity among the included studies, as evidenced by I^2^ values ranging from 75 to 91%, indicating that differences in study populations, methodologies, and interventions strongly influenced the summary effects. Correspondingly, the wide prediction intervals for the overall effect sizes suggested considerable uncertainty about the effect size in future studies, reflecting the substantial heterogeneity among the existing studies. While the overall findings indicate significant improvements in PPD reduction and CAL gain with the adjunctive use of APCs, the variability necessitates cautious interpretation.

The risk of bias assessment indicated a spectrum of methodological quality among the studies included in this systematic review (Fig. 7). The ambiguity largely stemmed from inadequate reporting of random sequence generation, allocation sequence procedures, and the absence of blinding for both participants and outcome assessors. The majority of the included studies failed to present pre-registered trial protocols [32, 34, 35, 43, 45, 46] or reported registry numbers that did not correspond with the published study [47]. Furthermore, the asymmetry observed in the funnel plots raised concerns about potential publication bias, which could have led to an overestimation of the treatment effects in the present meta-analysis. However, while the asymmetry might indicate publication bias, it could also be influenced by the heterogeneity among the included studies, the variations in the study quality, differences in study methodologies, or chance. Future studies should ensure detailed and transparent reporting of randomization procedures, enhancement of blinding practices, and adopt comprehensive publicly accessible pre-registered protocols adhering to established guidelines such as CONSORT [64], thereby increasing the reliability of the findings and improving the overall quality of the research.

This systematic review has several limitations that should be considered when interpreting the findings. The potential for publication bias, which we accounted for when assessing the certainty of the evidence, could have influenced the results. Despite the comprehensive search strategy, some relevant studies may have been omitted, and while the data extraction was performed meticulously, the possibility of errors can not be entirely eliminated. The heterogeneity among the included studies, with variations in populations and interventions, including variations in the preparation of APCs, presented data synthesis and interpretation challenges. The overall quality of the included studies varied, with most exhibiting an unclear risk of bias, which may affect the reliability of the conclusions, and the included studies had relatively small sample sizes, which limits the precision of the findings and may have contributed to the imprecision of the overall results.

Furthermore, the preparation protocols for APCs are not universally standardized, which presents challenges in comparing clinical outcomes across studies and optimizing therapeutic applications. While general preparation principles exist, protocols vary due to equipment, clinical objectives, and differences in commercial systems. While efforts such as the Dohan Ehrenfest classification and consensus guidelines by the International Cellular Medicine Society have sought to standardize APC preparation by defining platelet concentration thresholds and cellular content [60], biological variability among patients, the volume of blood used to generate the APCs, and differences in activation methods contribute to the lack of uniformity. These inconsistencies underscore the need for rigorous documentation and adherence to published protocols in research and clinical practice to improve reproducibility and comparability.

## Conclusion

Despite its limitations, this systematic review provides a comprehensive synthesis of the available evidence and highlights areas for future research. The use of APCs, regardless of their subtype, appears to provide additional benefits in PPD reduction and CAL gain compared to NSPT alone. When applying these findings to practice, clinicians should consider patient-specific factors and clinical contexts. Creating standardized APC preparation protocols, followed by additional studies with consistent research protocols and reporting methodologies could significantly strengthen the evidence base, providing more straightforward guidance for clinical decision-making in applying APCs to NSPT.

## Supplementary Information

Below is the link to the electronic supplementary material.

Appendix 1 (DOCX 30.5 KB)

Appendix 2 (DOCX 31.5 KB)

Appendix 3 (DOCX 868 KB)

## Data Availability

The data that support the findings of this study are available from the corresponding author upon reasonable request.
