# Supplementary material for: Autologous platelet concentrates as adjuncts to non-surgical periodontal therapy: a systematic review and meta-analysis
Source: Clin Oral Investig. 2025 Jan 22;29(1):74. doi: 10.1007/s00784-024-06128-w (PMC11754314; doi:10.1007/s00784-024-06128-w)
Supplement: Supplementary file 2 — (DOCX 31.5 KB) [file 784_2024_6128_MOESM2_ESM.docx]

**APPENDIX 2**

Search Strategies

| ID | **MEDLINE (OVID)** <1946 to February 24, 2024> | Results |
| --- | --- | --- |
| 1. | exp Periodontitis/ | 35695 |
| 2. | (periodontiti* or pericementitid* or pericementiti* or "gum* diseas*" or "gum* bleed*" or "periodont* diseas*").mp. | 76102 |
| 3. | 1 or 2 | 80168 |
| 4. | exp Dental Scaling/ | 4686 |
| 5. | ("dent* scal*" or "root* scal*" or "subging* scal*" or "sub gingiv* scal*" or "supraging* scal*" or "supra ging* scal*").mp. | 4685 |
| 6. | exp "Root Planing"/ 2145 | 2145 |
| 7. | "root* plan*".mp. | 5067 |
| 8. | exp Subgingival Curettage/ | 2987 |
| 9. | ("subging* curettag*" or "sub ging* curretag*" or "root* debridement*").mp. | 1141 |
| 10. | (curettag* adj4 (ging* or "sub ging*" or "sub-ging*" or subging*)).mp. | 1051 |
| 11. | (debridement* adj4 (periodont* epitheli* or "root* surface*" or "full mouth*" or "dent* quadrant*")).mp. | 201 |
| 12. | exp Dental Prophylaxis/ | 8163 |
| 13. | (prophylaxis adj4 (dent* or teeth or tooth or oral)).mp. | 7627 |
| 14. | exp Dental Deposits/ | 21465 |
| 15. | (deposit* adj4 (tooth or teeth or oral)).mp. | 549 |
| 16. | (dent* adj3 (plaque or calculus or tartar)).mp. | 26022 |
| 17. | exp Dental Polishing/ | 2695 |
| 18. | (polish* adj4 (dent* or tooth or teeth)).mp. | 3575 |
| 19. | (mechanic* adj3 debridement*).mp. | 904 |
| 20. | (instrument* adj3 ("supra ging*" or supraging* or "sub ging*" or subging* or "full mouth*")).mp. | 168 |
| 21. | exp Dental Instruments/ | 6789 |
| 22. | ("root* instrument*" or "manual instrument*" or "hand* instrument*" or "power instrument*").mp. | 1560 |
| 23. | ("periodont* therap*" or "non surgic* periodont* therap").mp. | 5485 |
| 24. | exp Dentistry/ | 438689 |
| 25. | dental.mp. | 527038 |
| 26. | 24 or 25 | 650920 |
| 27. | (sonic* or ultrasonic* or "ultra sonic*" or oscillat* or reciprocat* or rotat* or diamond* or perioplan* or rootsharp* or "power driven" or curette* or scaler*).mp. | 503896 |
| 28. | 26 and 27 | 15289 |
| 29. | or/4-23 | 55176 |
| 30. | 28 or 29 | 66692 |
| 31. | 3 and 30 | 17246 |
| 32. | limit 31 to (clinical trial, all or clinical trial or randomized controlled trial) | 2568 |
| 33. | (((single adj (blind* or masked)) or double) adj (blind* or masked)).ab. or (((single adj (blind* or masked)) or double) adj (blind* or masked)).ti | 178230 |
| 34. | (randomized or randomly or placebo or trial or (controlled adj study)).ab. or (randomized or randomly or placebo or trial or (controlled adj study)).ti. | 1560232 |
| 35. | (randomized controlled trial or controlled clinical trial).pt. or randomized.ab. or placebo.ab. or drug therapy.fs. or randomly.ab. or trial.ab. or groups.ab. | 5888497 |
| 36. | 33 or 34 or 35 | 5942385 |
| 37. | 31 and 36 | 6848 |
| 38. | 32 or 37 | 6952 |
| 39. | limit 38 to humans | 5841 |
| 40. | limit 38 to animals | 440 |
| 41. | 38 not 40 | 6512 |
| 42. | limit 41 to adult | 3147 |
| 43. | exp Blood Platelets/ | 83059 |
| 44. | exp Platelet-Derived Growth Factor/ | 14398 |
| 45. | ("Platelet* preparation*" or "Platelet* concentrate*" or "Autologous serum*" or "autologous platelet* concentrate*").mp. | 6860 |
| 46. | 43 or 44 or 45 | 101357 |
| 47. | exp Platelet-Rich Plasma/ | 7057 |
| 48. | ("platelet* rich plasma" or "platelet*-rich plasma" or PRP*).mp. | 33092 |
| 49. | exp Platelet-Rich Fibrin/ | 1027 |
| 50. | ("platelet* rich fibrin*" or "platelet*-rich fibrin*" or PRF*).mp. | 8102 |
| 51. | (iPRF* or i-PRF* or "injectable PRF*" or "injectable-PRF*" or "injectable platelet* rich fibrin*" or "injectable platelet*-rich fibrin*").mp. | 229 |
| 52. | (APRF* or A-PRF* or "advanced PRF*" or "advanced-PRF*" or "advanced platelet* rich fibrin*" or "advanced platelet*-rich fibrin*").mp. | 518 |
| 53. | (TPRF* or T-PRF* or "titanium PRF*" or "titanium-PRF*" or "titanium prepared PRF*" or "titanium-prepared PRF*" or "titanium-prepared platelet*-rich fibrin*" or "titanium prepared platelet* rich fibrin*").mp. | 60 |
| 54. | (CGF* or "concentrated growth factor*").mp. | 942 |
| 55. | or/46-54 | 138058 |
| 56. | 39 and 55 | 57 |
| 57. | 42 and 55 | 33 |

| ID | **Embase (OVID)** <1980 to February 24, 2024> | Results |
| --- | --- | --- |
| 1. | exp Periodontitis/ | 54065 |
| 2. | (periodontiti* or pericementitid* or pericementiti* or “gum* diseas*” or “gum* bleed*” or “periodont* diseas*”).mp. | 96057 |
| 3. | 1 or 2 | 106610 |
| 4. | exp Dental Scaling/ | 1452 |
| 5. | (“dent* scal*” or “root* scal*” or “subging* scal*” or "sub gingiv* scal*" or “supraging* scal*” or “supra ging* scal*”).mp. | 2147 |
| 6. | exp Root Planing/ | 1236 |
| 7. | "root* plan*".mp. | 4599 |
| 8. | exp Subgingival Curettage/ | 161 |
| 9. | ("subging* curettag*" or "sub ging* curretag*" or "root* debridement*").mp. | 203 |
| 10. | (curettag* adj4 (ging* or "sub ging*" or "sub-ging*" or subging*)).mp. | 126 |
| 11. | (debridement* adj4 (periodont* epitheli* or "root* surface*" or "full mouth*" or "dent* quadrant*")).mp. | 193 |
| 12. | exp Dental Prophylaxis/ | 3062 |
| 13. | (prophylaxis adj4 (dent* or teeth or tooth or oral)).mp. | 6043 |
| 14. | exp Dental Deposits/ | 23098 |
| 15. | (deposit* adj4 (tooth or teeth or oral)).mp. | 605 |
| 16. | (dent* adj3 (plaque or calculus or tartar)).mp. | 9317 |
| 17. | exp Dental Polishing/ | 233 |
| 18. | (polish* adj4 (dent* or tooth or teeth)).mp. | 1420 |
| 19. | (mechanic* adj3 debridement*).mp. | 1160 |
| 20. | (instrument* adj3 ("supra ging*" or supraging* or "sub ging*" or subging* or "full mouth*")).mp. | 194 |
| 21. | exp Dental Instruments/ | 137980 |
| 22. | ("root* instrument*" or "manual instrument*" or "hand* instrument*" or "power instrument*").mp. | 1675 |
| 23. | ("periodont* therap*" or "non surgic* periodont* therap").mp. | 5445 |
| 24. | exp Dentistry/ | 109637 |
| 25. | dental.mp. | 422410 |
| 26. | 24 or 25 | 475009 |
| 27. | (sonic* or ultrasonic* or "ultra sonic*" or oscillat* or reciprocat* or rotat* or diamond* or perioplan* or rootsharp* or "power driven" or curette* or scaler*).mp. | 564522 |
| 28. | 26 and 27 | 13037 |
| 29. | or/4-23 | 175944 |
| 30. | 28 or 29 | 183054 |
| 31. | 3 and 30 | 32259 |
| 32. | limit 31 to randomized controlled trial | 3518 |
| 33. | (((single adj (blind* or masked)) or double) adj (blind* or masked)).ab. or (((single adj (blind* or masked)) or double) adj (blind* or masked)).ti. | 254112 |
| 34. | (randomized or randomly or placebo or trial or (controlled adj study)).ab. or (randomized or randomly or placebo or trial or (controlled adj study)).ti. | 2227412 |
| 35. | (randomized controlled trial or controlled clinical trial).pt. or randomized.ab. or placebo.ab. or drug therapy.fs. or randomly.ab. or trial.ab. or groups.ab. | 8824903 |
| 36. | 33 or 34 or 35 | 8911734 |
| 37. | 31 and 36 | 11995 |
| 38. | 32 or 37 | 12164 |
| 39. | limit 38 to humans | 11256 |
| 40. | limit 38 to animals | 659 |
| 41. | 38 not 40 | 11505 |
| 42. | limit 41 to adult | 6241 |
| 43. | exp Blood Platelets/ | 128617 |
| 44. | exp Platelet-Derived Growth Factor/ | 39526 |
| 45. | ("Platelet* preparation*" or "Platelet* concentrate*" or "Autologous serum*" or "autologous platelet* concentrate*").mp. | 10819 |
| 46. | 43 or 44 or 45 | 174395 |
| 47. | exp Platelet-Rich Plasma/ | 20361 |
| 48. | ("platelet* rich plasma" or "platelet*-rich plasma" or PRP*).mp. | 42156 |
| 49. | exp Platelet-Rich Fibrin/ | 2000 |
| 50. | ("platelet* rich fibrin*" or "platelet*-rich fibrin*" or PRF*).mp. | 11206 |
| 51. | (iPRF* or i-PRF* or "injectable PRF*" or "injectable-PRF*" or "injectable platelet* rich fibrin*" or "injectable platelet*-rich fibrin*").mp. | 215 |
| 52. | (APRF* or A-PRF* or "advanced PRF*" or "advanced-PRF*" or "advanced platelet* rich fibrin*" or "advanced platelet*-rich fibrin*").mp. | 599 |
| 53. | (TPRF* or T-PRF* or "titanium PRF*" or "titanium-PRF*" or "titanium prepared PRF*" or "titanium-prepared PRF*" or "titanium-prepared platelet*-rich fibrin*" or "titanium prepared platelet* rich fibrin*").mp. | 72 |
| 54. | (CGF* or "concentrated growth factor*").mp. | 1251 |
| 55. | or/46-54 | 225563 |
| 56. | 39 and 55 | 142 |
| 57. | 42 and 55 | 94 |

| ID | **Cochrane** <24.2.2024> | Results |
| --- | --- | --- |
| #1 | MeSH descriptor: [Periodontitis] explode all trees | 3913 |
| #2 | MeSH descriptor: [Aggressive Periodontitis] explode all trees | 139 |
| #3 | MeSH descriptor: [Chronic Periodontitis] explode all trees | 1110 |
| #4 | MeSH descriptor: [Periodontal Abscess] explode all trees | 20 |
| #5 | MeSH descriptor: [Periodontal Pocket] explode all trees | 1441 |
| #6 | MeSH descriptor: [Alveolar Bone Loss] explode all trees | 1686 |
| #7 | periodont* | 17857 |
| #8 | periodont* NEAR/1 “attachment loss” | 1075 |
| #9 | periodont* NEAR/1 pocket* | 2782 |
| #10 | alveolar NEAR/1 (loss or atrophy*) | 422 |
| #11 | (periodont* OR alveolar) NEAR/2 resorption | 193 |
| #12 | #1 OR #2 OR #3 OR #4 OR #5 OR #6 OR #7 OR #8 OR #9 OR #10 OR #11 | 18618 |
| #13 | MeSH descriptor: [Dental Scaling] explode all trees | 1522 |
| #14 | MeSH descriptor: [Subgingival Curettage] explode all trees | 1010 |
| #15 | MeSH descriptor: [Periodontal Debridement] explode all trees | 129 |
| #16 | MeSH descriptor: [Root Planing] explode all trees | 911 |
| #17 | root NEXT plan* | 2897 |
| #18 | (periodont* OR supragingival OR supra-gingival OR subgingival OR sub-gingival OR “supra gingival” OR “sub gingival”) NEXT/1 (debrid* OR scal* OR curett*) | 905 |
| #19 | professional NEAR/1 “plaque removal” | 21 |
| #20 | PMPR | 22 |
| #21 | #13 OR #14 OR #15 OR #16 OR #17 OR #18 OR #19 OR #20 | 3738 |
| #22 | #12 AND #21 | 3512 |
| #23 | MeSH descriptor: [Blood Platelets] explode all trees | 2517 |
| #24 | MeSH descriptor: [Serum] explode all trees | 1073 |
| #25 | platelet* OR (platelet* concentrate*) OR (platelet* preparation*) OR (autologous serum*) OR APC* OR (autologous platelet* concentrate*) | 39445 |
| #26 | #23 OR #24 OR #25 | 40424 |
| #27 | MeSH descriptor: [Platelet-Rich Plasma] explode all trees | 1188 |
| #28 | PRP* OR (platelet* rich plasma*) | 5177 |
| #29 | #27 OR #28 | 5463 |
| #30 | MeSH descriptor: [Platelet-Rich Fibrin] explode all trees | 305 |
| #31 | PRF* OR (platelet* rich fibrin*) | 2218 |
| #32 | iPRF* OR i-PRF* OR (injectable PRF*) OR (injectable platelet* rich fibrin*) | 224 |
| #33 | APRF* OR A-PRF* OR (advanced platelet* rich fibrin*) | 305 |
| #34 | TPRF* OR T-PRF* OR (titanium prepared platelet* rich fibrin*) OR (titanium platelet* rich fibrin*) | 73 |
| #35 | #30 OR #31 OR #32 OR #33 OR #34 | 2224 |
| #36 | CGF* OR (concentrated growth factor*) | 622 |
| #37 | #29 OR #35 OR #36 | 7602 |
| #38 | #22 AND #37 in Trials | 87 |
| **MedNar** <February 26, 2024> | | Results |
| (periodont* diseas* AND (debrid* OR scal* OR curett* OR "non-surg* perio* therap*" OR NSPT OR PMPR OR "professional mechanic* plaque removal*")) AND ("platelet* concentrate*" OR PRP* OR "platelet* rich plasma*" OR PRF* OR IPRF* OR "injectable PRF*" OR "injectable platelet* rich fibrin*" OR "injectable PRF*" OR "platelet* rich fibrin*" OR APRF* OR "advanced PRF*" OR "advanced platelet* rich fibrin*" OR TPRF* OR "titanium PRF*" OR "titanium prepared platelet* rich fibrin*" OR CGF* OR "concentrated growth factor*") | | 286 |
| Refine by: Clinical Trials | | 52 |

| **CORE** <February 26, 2024> | Results |
| --- | --- |
| (periodont* diseas* AND (debrid* OR scal* OR curett* OR "non-surg* perio* therap*" OR NSPT OR PMPR OR "professional mechanic* plaque removal*")) AND ("platelet* concentrate*" OR PRP* OR "platelet* rich plasma*" OR PRF* OR IPRF* OR "injectable PRF*" OR "injectable platelet* rich fibrin*" OR "injectable PRF*" OR "platelet* rich fibrin*" OR APRF* OR "advanced PRF*" OR "advanced platelet* rich fibrin*" OR TPRF* OR "titanium PRF*" OR "titanium prepared platelet* rich fibrin*" OR CGF* OR "concentrated growth factor*") | 3633 |
| Field: Medicine | 164 |
| Type: Research | 63 |
