# Supplementary material for: Autologous platelet concentrates as adjuncts to non-surgical periodontal therapy: a systematic review and meta-analysis
Source: Clin Oral Investig. 2025 Jan 22;29(1):74. doi: 10.1007/s00784-024-06128-w (PMC11754314; doi:10.1007/s00784-024-06128-w)
Supplement: Supplementary file 3 — (DOCX 868 KB) [file 784_2024_6128_MOESM3_ESM.docx]

**APPENDIX 3**


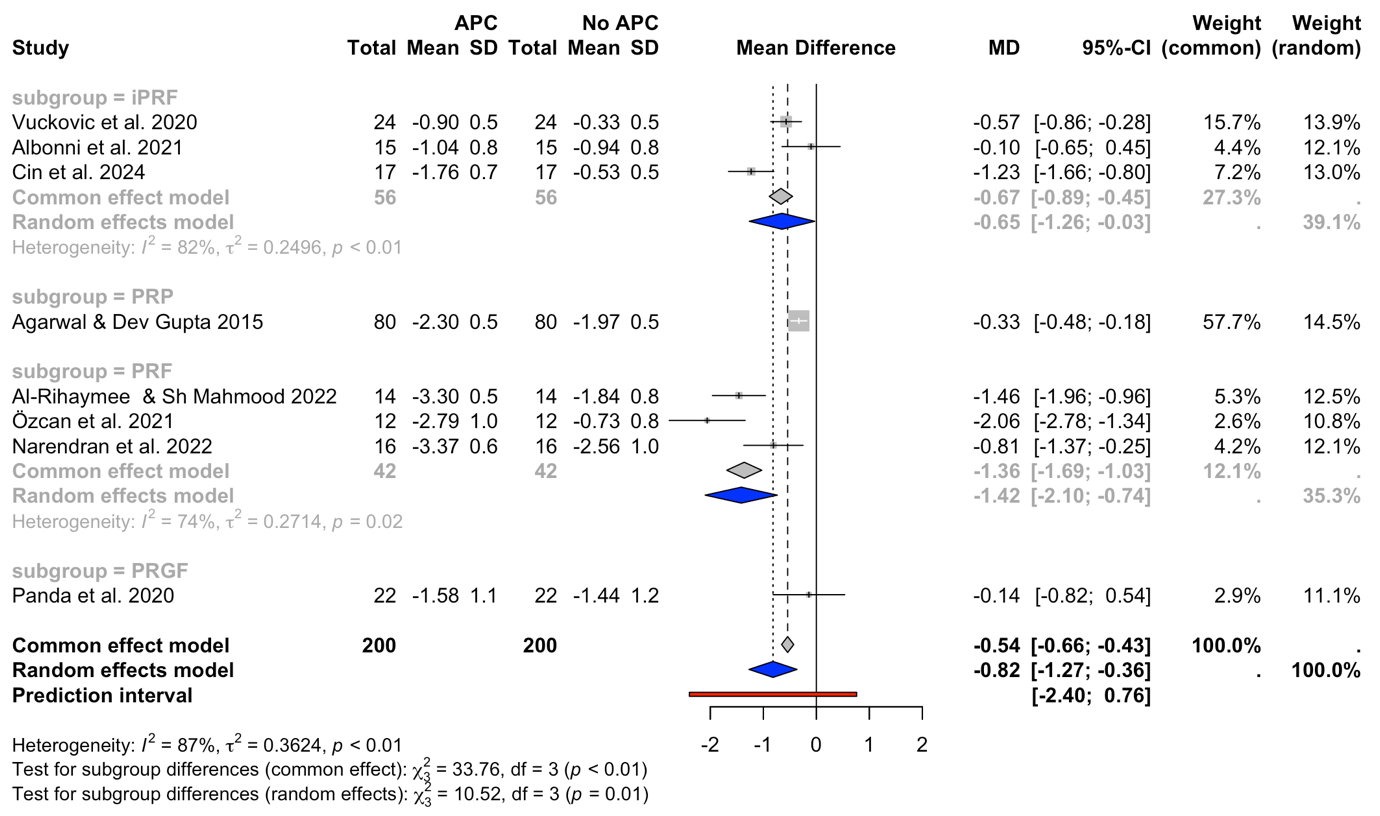
**
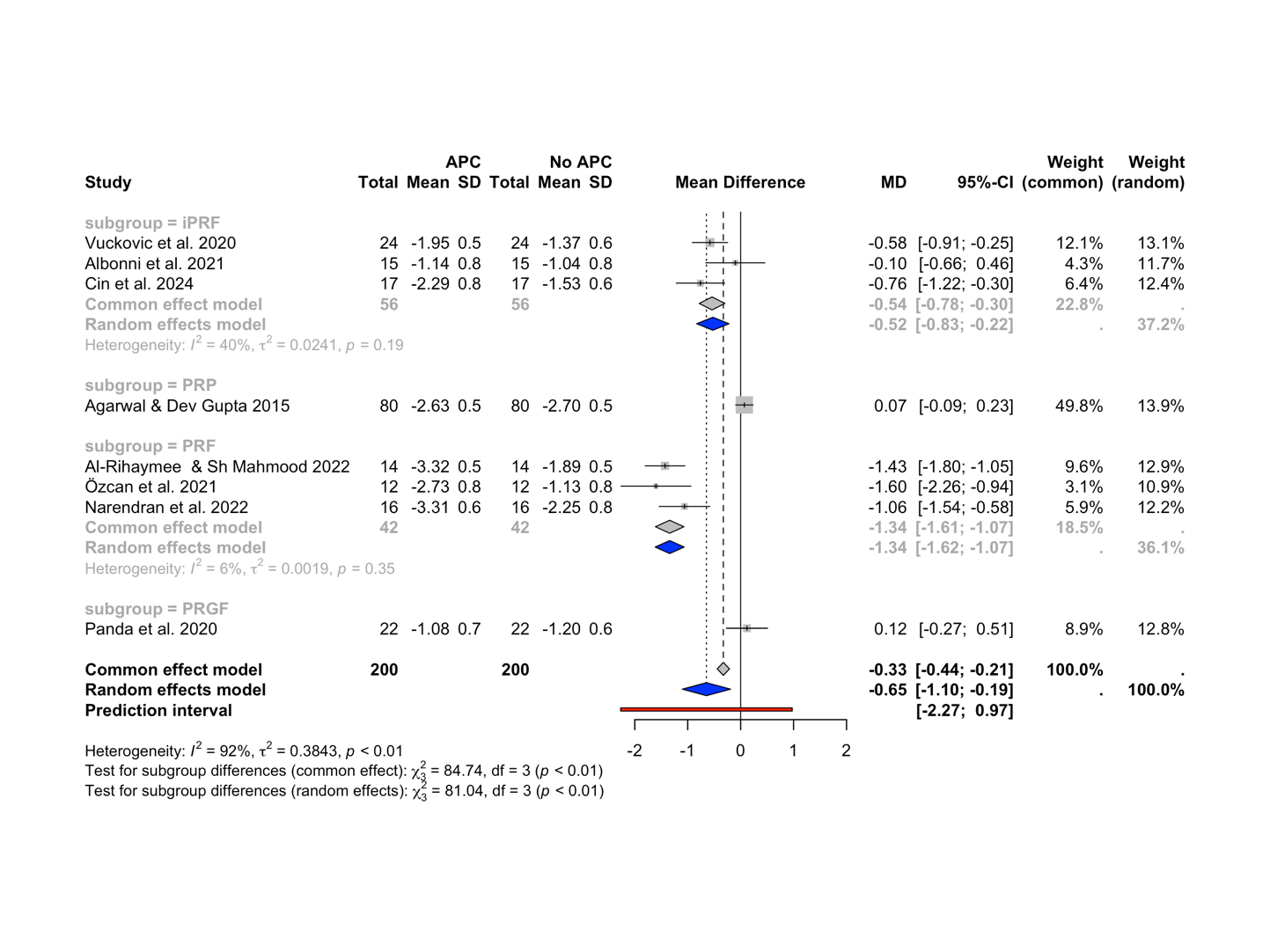
**Results of sensitivity analyses

**Figure 10.** CAL change at the 3-month follow-up in studies with a single APC application and low to unclear risk of bias.

**Figure 9.** PPD change at the 3-month follow-up in studies with a single APC application and low to unclear risk of bias.


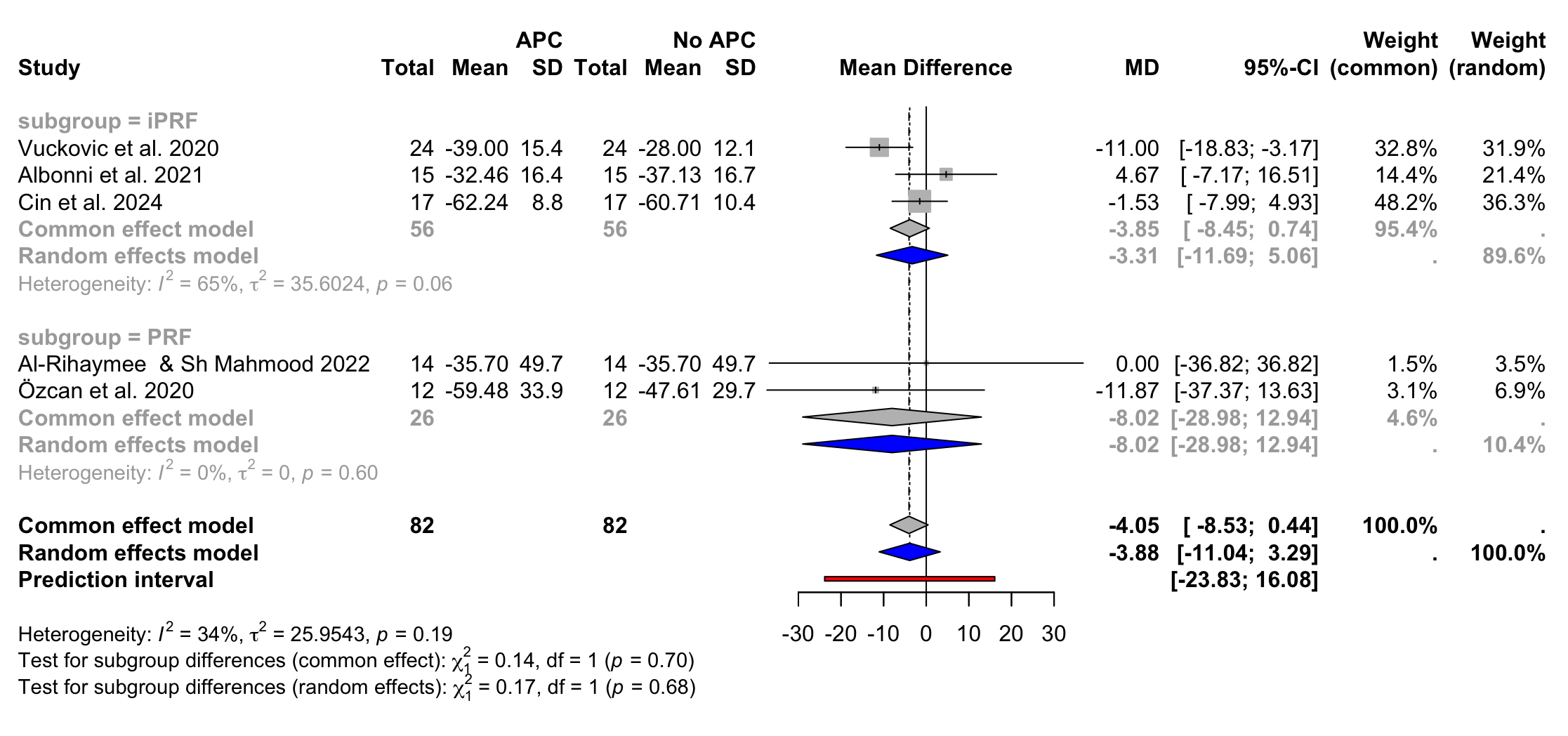


**Figure 11.** BoP changes at the 3-month follow-up in studies with a single APC application and low to unclear risk of bias.
